# Short-Term Effect of Auditory Stimulation on Neural Activities: A Scoping Review of Longitudinal Electroencephalography and Magnetoencephalography Studies

**DOI:** 10.3390/brainsci14020131

**Published:** 2024-01-26

**Authors:** Kanon Kobayashi, Yasushi Shiba, Shiori Honda, Shinichiro Nakajima, Shinya Fujii, Masaru Mimura, Yoshihiro Noda

**Affiliations:** 1 Department of Neuropsychiatry, Keio University School of Medicine, 35 Shinanomachi, Shinjuku-ku, Tokyo 160-8582, Japan; kanon.kk.kobayashi@keio.jp (K.K.); shiori.0913.honda@keio.jp (S.H.);; 2 Faculty of Medicine, University of Tokyo, Tokyo 113-8655, Japan; shigecc.88@gmail.com; 3 Faculty of Environment and Information Studies, Keio University, Fujisawa 252-0816, Japan

**Keywords:** auditory training, auditory stimuli, neural oscillations, functional connectivity, predictive coding, temporal expectation, attention, neuroplasticity

## Abstract

Explored through EEG/MEG, auditory stimuli function as a suitable research probe to reveal various neural activities, including event-related potentials, brain oscillations and functional connectivity. Accumulating evidence in this field stems from studies investigating neuroplasticity induced by long-term auditory training, specifically cross-sectional studies comparing musicians and non-musicians as well as longitudinal studies with musicians. In contrast, studies that address the neural effects of short-term interventions whose duration lasts from minutes to hours are only beginning to be featured. Over the past decade, an increasing body of evidence has shown that short-term auditory interventions evoke rapid changes in neural activities, and oscillatory fluctuations can be observed even in the prestimulus period. In this scoping review, we divided the extracted neurophysiological studies into three groups to discuss neural activities with short-term auditory interventions: the pre-stimulus period, during stimulation, and a comparison of before and after stimulation. We show that oscillatory activities vary depending on the context of the stimuli and are greatly affected by the interplay of bottom-up and top-down modulational mechanisms, including attention. We conclude that the observed rapid changes in neural activitiesin the auditory cortex and the higher-order cognitive part of the brain are causally attributed to short-term auditory interventions.

## 1. Introduction

Recent advances in Electroencephalography (EEG) and magnetoencephalography (MEG) reconstruction methods enable detailed mapping of phase interactions, revealing extensive cortical networks with significant behavioral relevance [1]. EEG and MEG studies reveal that neural oscillatory activities can affect perceptual processing and cognitive operations, setting the stage for understanding how the neural system interacts with external information [2]. Temporal coordination of distributed neuronal activities, known as neural synchrony, serves as a mechanism for feature integration in neuronal assemblies [3]. Oscillatory entrainment refers to the synchronization of two or more self-sustained oscillators, which are systems capable of autonomously generating their own rhythms [4]. Entrainment of neural rhythms to external stimuli has long been hypothesized to underlie sensory detection and discrimination [5,6]. Entrainment of rhythmic activities works in favor of controlling sensory gain and selecting task-relevant information [7]. Recent studies showed that periodic fluctuations in sensory sampling are also governed by intrinsic spontaneous oscillations [8]. In other words, oscillations of neural activities exist as endogenous rhythms even in the absence of external stimuli [9]. 

Over the years, auditory stimuli have been used as suitable research probes for studying neural activities. The aforementioned techniques such as EEG and MEG are potent tools for measuring dynamic brain oscillations aligned with dynamic regularities in auditory stimuli [7]. For example, in 2000, Haenschel, et al. [10] observed an interdependent oscillation transition from the gamma to beta band in the auditory cortex in response to a novel stimulus using sequential pure tones in human EEG. The authors discussed that the interplay between these two oscillations is similar to that observed *in vitro* in hippocampal slice preparations in response to a novel stimulus presentation, which suggests that the auditory stimulus design can mimic the *in vitro* paradigm. This provides stronger evidence that actual auditory-evoked specific cortical activities can be measured noninvasively. A more recent EEG/MEG study showed that the rhythmic regularity of sound sequences modulates oscillatory activities in the delta and beta band in the auditory cortex and facilitates perceptual processing [11]. When researchers employ EEG/MEG to investigate auditory processing, they can infer functional connectivity by detecting the auditory-evoked neural synchrony [1]. Functional connectivity reflects statistical relationships between spatially distant brain regions by assessing the temporal coincidence of neurophysiological events when they are correlated in functional behavior, and provides direct analysis of dynamic brain networks [12]. Thus, neurophysiological studies using sound stimuli have helped to elucidate the intrinsic brain rhythm, as well as its entrainment to external stimuli and the connectivity formed in two different regions.

Long-term auditory stimulation has traditionally been used to evoke neuronal responses. Musicians show enlarged auditory cortical-evoked potentials to piano tones [13,14], and this effect can be additionally modulated according to the timbre of their own musical instrument [15,16]. These studies recruited musicians with a history of long-term musical discipline, or participants without musical background trained for several months to years. Many studies compared those people with long-term musical exposure to non-musicians in a cross-sectional design, to assess the differences in their brain responses to auditory stimuli (e.g., [17,18,19]). However, from the results of cross-sectional studies, it cannot be determined whether the excellence of musicians’ auditory skills should be attributed to their innate capabilities and inherent traits, or neuroplastic effects acquired by the exposure to music [20,21,22].

In contrast to cross-sectional studies, longitudinal studies are expected to capture the effects of acquired skills because they observe differences before and after long-term musical training with the same population [23,24]. For instance, in a longitudinal study of non-musician children who received six months of music training, pre-and post-training pitch discrimination tasks with an EEG recording revealed that after the training the children showed an improvement in pitch discrimination ability and an increase in the amplitude of the N300 component [25]. Here, through the longitudinal design, the authors made sure that they ruled out the possibilities of preexisting differences in perceptual and cognitive capabilities among participants. Nowadays, longitudinal studies are considered more and more important to assess the causal relationship between auditory interventions and neuroplasticity [15,26].

On the other hand, there is a line of studies using brief interventions with auditory stimuli, whose duration lasts from minutes to hours, to cause changes in brain responses [26,27]. For example, Pantev, et al. [28] reported that within as short as three hours of listening to auditory stimuli that had been band-pass filtered to remove specific frequencies, neuronal responses to tones within the filter bandwidth were reduced. Indeed, the effects of auditory training on the brain are extremely quick, with some effects occurring within several minutes of the start of training [29,30,31]. This kind of studies on neural oscillations with such short-term interventions has only begun to be addressed in the past ten years. 

The development of an experimental paradigm targeting top-down brain mechanisms has also been remarkable over the decade. While there was substantial evidence for neural entrainment as an automatic, bottom-up response before 2010, only a few attempts have been made to address its top-down modulation [30,32,33]. It is becoming increasingly clear, from recent studies, that neural oscillations and entrainment may be core ingredients of higher-order cognitive processes such as attention, memory, and awareness, and that they are intertwined with low-level sensory processing [34,35,36]. In their review published in 2011, Pantev and Herholz [15] pointed out the need for further research to explore the effect of short-term auditory training on attention and other higher cognitive abilities. In this review, we summarize the growing evidence of these bidirectional processes of bottom-up and top-down processing since 2011, which is investigated through neurophysiological studies using sound stimuli.

It is noteworthy that participants are not required to have a musical background in order to study the direct effect of musical training through longitudinal studies; there is more evidence that musical novices are capable of learning some rules of musical stimuli to which they are exposed [37,38,39]. For example, an EEG study by Koelsch, et al. [37] provided evidence that music novices are sensitive to deviations from chord rules, suggesting the existence of tacit knowledge about musical rules they have. Other than chords, there is evidence that non-musicians are sensitive to key elements of music, such as tonality [34,40], meter [41], and melody [42].

Another important point to note is the fact that the oscillations can fluctuate depending on the context. On the examination of short-term dynamic processing of auditory stimuli, the fluctuation of the neural oscillations during the prestimulus period is an important research subject [35,43,44]. While participants are waiting for the auditory target stimuli to be presented, temporal expectation contributes to the predictive and preparatory state of the brain [45]. The effect of temporal expectation on neural activities have been investigated by using informative temporal structures, such as cues, varied intervals between stimuli, and context regularity [46]. These structures manipulate the participants’ prediction by giving them information on the likelihood of an upcoming event at a given time. In some experiments, cues refer to the temporal probability distribution, the modality of the target, and the spatial orientation of the target [47,48]. The validity of the cues is also sometimes manipulated, with or without informing participants [49,50]. The investigations of this field are made possible by short-term and real-time measurement of auditory processing through electrophysiological studies. To be sure, neural responses in the prestimulus period are not those “elicited” by the actual auditory stimuli. However, during the prestimulus period, this kind of prediction about the specific factors of upcoming auditory stimuli is formed by the auditory listening condition itself. We focused on this point in this current review, based on the ground that prestimulus period fluctuations of oscillations formed in response to contextual factors have some influence on the following stimulus processing.

**Figure 1:**
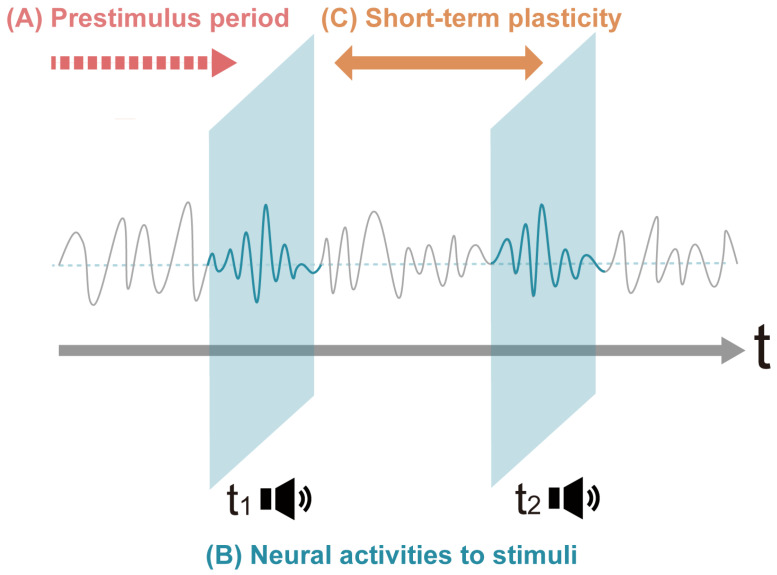
An overview of short-term neuroplastic effects through auditory interventions. Our review consists of three parts. (**A**) We first observe the fluctuation of the neural oscillations during the prestimulus period depending on the context. (**B**) Next, we focus on the neural responses during the auditory stimulation. We describe how the neural responses to the sound stimulus differ depending on the conditions. (**C**) In the final section, we show the neuroplastic changes before and after the short-term auditory interventions. The gray curved line represents the fluctuations in neural oscillatory activities. The loudspeaker signs represent a series of auditory stimulations. The horizontal axis represents the passage of time.

This scoping review provides an overview of the effect on neural mechanisms of short-term auditory interventions whose duration is minutes to hours, with participants recruited irrespective of previous musical training experience. Previous reviews have pointed out the significant effect of musical training [21,26,27], compiling comprehensive findings of studies with both short-term and long-term musical training. These reviews included musical training of not only listening but also playing instruments, musical imagery, or cross-modal training using auditory and other modalities. This makes the interpretations of the results in this area dissipative, as the interpretations of the results vary widely depending on the nature of the training. Therefore, we limited the training content to simply listening and thereby aimed to track the oscillatory changes evoked by auditory listening over a short period of time, using EEG or MEG to follow the time course of auditory processing. Specifically, the objectives of this scoping review are to examine prestimulus brain activities driven by the prediction of the upcoming stimuli (Figure 1A), neural responses to auditory stimuli during listening to pure sounds or musical stimuli (Figure 1B), and short-term neuroplastic changes before and after auditory interventions (Figure 1C).

## 2. Materials and Methods

### 2.1. Search Strategy

The review was performed using the Preferred Reporting Items for Systematic Reviews and Meta-Analyses (PRISMA) guidelines for scoping reviews [51]. The checklist for the PRISMA-ScR is in the Appendix A Table A1. Research articles examined for human subjects written in English were screened by two independent reviewers (K.K. and Y.S.) using PubMed from 15 February 2011 to 8 August 2022. In this way, we narrowed down the publication year because we wanted to scope out the latest findings in the past decade.

We aimed to know what kind of changes in brain responses or functional connectivity, namely plasticity, could be observed by using music and other sound stimuli as the interventions. We searched for experiments with neurophysiological methods of EEG and MEG, and the words oscillation, entrainment, or brain rhythm were likely keywords in observations using these techniques. This background led us to set the search terms as “((Music OR Auditory) AND (connectivity OR plasticity) AND ((Brain rhythm) OR oscillation OR entrainment OR neurophysiol* OR electroencephalog* OR EEG OR magnetoencephalog* OR MEG) NOT (NIRS OR SPECT OR PET))”.

In addition, relevant studies that were not identified in the initial search and should have been included were added through manual searches. The articles that were included in the final analysis were assessed for risk of bias according to the Risk of Bias Assessment Tool for Nonrandomized Studies (RoBANS) [52].

### 2.2. Selection Criteria

In this review, we aimed to explore generalizable and universal phenomena of neurophysiological changes evoked by short-term sound stimulation (Table 1). We double-checked the eligible criteria to examine methods of auditory interventions and selection of participants as follows:

Inclusion criteria:

(1) Studies that used auditory stimuli consisting of pure tones or music (we included studies that used visual or tactile cues for auditory stimuli and studies that manipulated attentional listening by showing silent films in parallel with auditory stimuli [53], as long as the main focus was on auditory modality); (2) studies with short-term interventions in which the duration of auditory training was minutes to hours; (3) longitudinal studies with healthy participants irrespective of age and past musical training experiences; (4) studies in which participants “listened to” stimuli in an “awake” state (we included studies involving the tasks of tapping along to auditory stimuli [54,55] because the focus of these studies was listening to auditory stimuli) and (5) studies whose neural activities were recorded by EEG or MEG.

Exclusion criteria:

(1) Studies that employed sentences, phonemes, syllables, combinations of music and other modalities for the presented stimuli or transcranial magnetic stimulation; (2) studies focusing on the long-term plastic effects of musical training over several weeks, months or years; (3) cross-sectional studies (e.g., studies that compared neurophysiological differences in processing sound with regard to some diseases, to the effect of aging and to professional musical training); (4) studies in which participants did not “listen to” stimuli in an “awake” state (e.g., studies that had participants play instruments, perform musical imagery or hear stimuli during sleep) or (5) studies that did not use electrophysiological measurements or studies that used recordings from implanted electrodes (we excluded these studies because those invasive devices were applied to patients, not to healthy people). 

**Table 1:** Selection criteria.

Conditions	Measures of Interest	Inclusion	Exclusion
Intervention, stimuli	Sound exposure	Pure tones Music White noise	Syllables Sentences Phonemes Crossmodal stimuli
Intervention, period	Short-term	Training over a few minutes, hours, days	Training over several months or years
Study design	Longitudinal	Monitoring a population over a certain period	Cross-sectional comparisons (musicians vs. non-musicians, different age groups, healthy vs. diseased)
Participants, subjects	Healthy people	People irrespective of age, diseases or musical skills	Patients
Participants, state	Awake and listening	Awake condition Attentive listening Passive listening	Playing instruments Vocalization Stimuli during sleep Musical imagery Listening combined with transcranial magnetic stimulation
Recording	Electrophysiological measures	MEG EEG	fMRI ECoG

## 3. Results

### 3.1. Overview of Studies

#### 3.1.1. Screening of Articles

**Figure 2:**
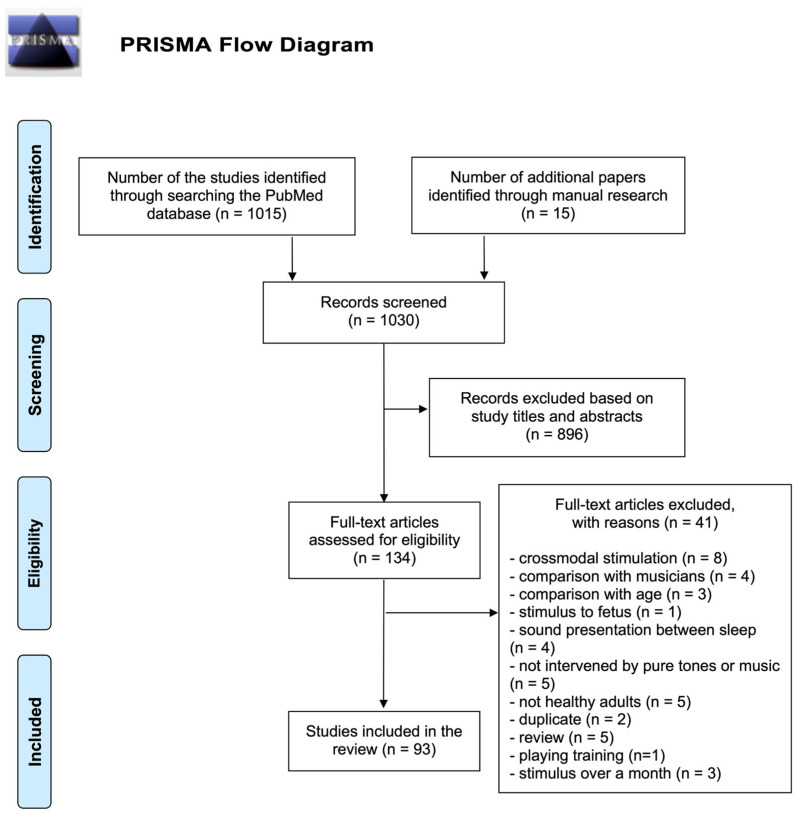
PRISMA Flow Chart. An initial search yielded 1015 articles and 15 articles were added through manual research. We carried out a check for eligibility using the procedure shown in the PRISMA Flow Chart. Through the assessment, 93 articles were selected.

An initial search yielded 1015 articles. This literature search also included 15 additional articles derived from manual searches. They were screened for eligibility using the procedure shown in the PRISMA Flow Chart (Figure 2). Two reviewers excluded 896 articles based on study titles and abstracts. For the remaining 134 articles, after a thorough review, 93 articles were finally determined to be eligible. According to RoBANS, four studies were considered to have high risk due to confounding variables and two studies with high risk due to selection of participants (see Appendix A Table A2 for details).

#### 3.1.2. Classification of Selected Articles

We classified the articles into three groups from a temporal perspective to provide an overview of the distribution of neurophysiological literature to date: (i) neural oscillations during the prestimulus period, (ii) neural responses to auditory stimuli during listening to pure sounds or musical stimuli, and (iii) short-term neuroplastic changes. We classified the articles into three groups from a temporal perspective to provide an overview of the distribution of the audio–neurophysiological literature to date.

First, eight articles that examined brain activities prior to stimuli exposure were classified as Group 1: Results 3.2.1. These studies measured EEG and/or MEG during the prestimulus period to determine the predictive state of the brain for the upcoming stimuli.

Secondly, 77 articles that examined responses during auditory processing were included in Group 2: Results 3.2.2. We further divided the studies in Group 2 into three subgroups based on methodology: 30 studies which measured various event-related potential (ERP) components (Group 2A: the Pure Tone Sequences section), 18 studies which compared neurophysiological responses to original and modified auditory stimuli (Group 2B: the Modification of Temporal Structure section) and 29 studies which controlled listening conditions and showed how auditory perception and cognition are separated or entwined (Group 2C: the Lower- and Higher-Order Functions in Representation of Auditory Objects section). 

Finally, eight studies that examined changes in neurophysiological activities before and after stimulation were classified into Group 3: Results 3.2.3. These studies demonstrated short-term neuroplastic changes by measuring neural activities before and after auditory stimulation. 

**Figure 3:**
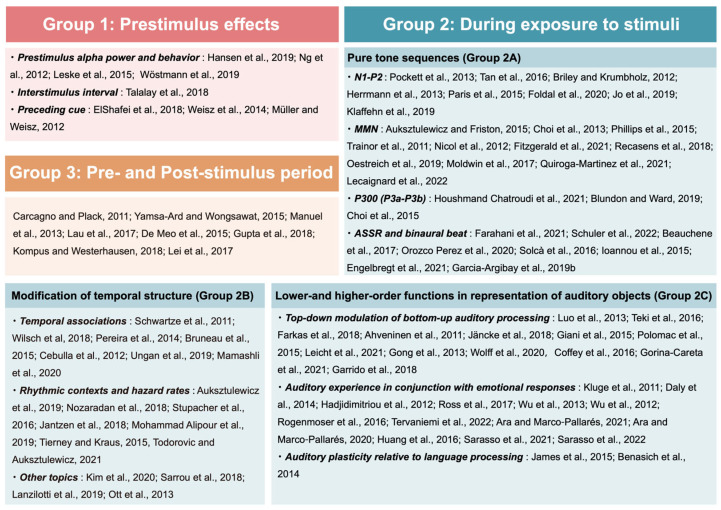
A summary of the categories of reviewed evidence. The screened articles included in the scoping review were classified into three groups in the temporal perspective. Group 1 includes eight articles that examined brain activities prior to stimuli exposure, revealing the predictive state for the upcoming stimuli processing. Group 2 includes 77 articles that examined neurophysiological responses during auditory processing. Group 3 includes eight articles that examined changes in neurophysiological activities before and after stimulation. Articles cited in more than one section are mentioned in this figure in the section where they appear for the first time [56,57,58,59,60,61,62,63,64,65,66,67,68,69,70,71,72,73,74,75,76,77,78,79,80,81,82,83,84,85,86,87,88,89,90,91,92,93,94,95,96,97,98,99,100,101,102,103,104,105,106,107,108,109,110,111,112,113,114,115,116,117,118,119,120,121,122,123,124,125,126,127,128,129,130,131,132,133,134,135,136,137,138,139,140,141,142,143].

These categories are not intended to be comprehensive or mutually exclusive, but the categorized evidence lays the groundwork for developing unified principles of neurophysiological effects. Figure 3 summarizes the study categories covered in this review, and Table 2 summarizes the articles included in the final analysis.

#### 3.1.3. Characteristics of the Interventions in the Selected Articles

Most studies examined changes in neural activities before and after interventions lasting from several minutes to hours, and several training sessions lasted for a total of a few hours over a week or a month [75,136,137,139,143]. The instructions to participants in those studies were asking them to listen to the presented stimuli. Other experiments included detecting target stimuli by exerting attention, doing some cognitive tasks that involve working memory (WM) and intelligence, discriminating among multiple sensory inputs or tapping along to the temporally regular rhythm. The index for behavioral performance was typically assessed via reaction time and accuracy of the judgments.

### 3.2. Individual Study Results and Synthesis

#### 3.2.1. Prestimulus Effects 1. Prestimulus Alpha Power and Behavior

Although two studies in the scope have reported the involvement of the prestimulus EEG phase of entrained oscillations with better performance in pitch discrimination [56,57], a conflicting idea is that a decrease in prestimulus alpha power, while predicting the upcoming target, correlates with the facilitatory processing of the following stimulus. Leske, et al. [58] supported this idea by showing that prestimulus alpha power was suppressed when threshold tones were correctly detected.

Here, increased task accuracy associated with the decrease in alpha activity may not be due to enhanced perceptual sensitivity. Rather, lower prestimulus alpha power has been shown to surface in the perceiver’s higher confidence in stimulus discrimination [59]. Note that the relationship between power and perception may be nonlinear. One study selected reported the opposite effect, where participants could successfully discriminate targets when the tone patterns were associated with increased alpha power [56]. The relationship between alpha power and perception will further be discussed in the Discussion 4.1.

1. Interstimulus Interval

One conservative method employed to investigate the prestimulus network is to manipulate the interval between sounds, i.e., the interstimulus interval (ISI). Altering ISIs provides a way to study the temporal expectations formed in a temporally uncertain environment. As these intervals or foreperiods vary between trials, participants have difficulty predicting the stimulus onset [58]. If the interval between sounds among trials is not constant, one cannot expect the upcoming stimulus to come after a certain time, and thus, uncertainty emerges as to the temporal appearance of the next stimulus. The attention at work which makes one learn when the next stimulus occurs from the condition probability is implicit expectation.

In the variable foreperiod condition, different prestimulus alpha power modulation patterns within the left and right auditory cortex were revealed. Leske, et al. [58] observed that the right A1 showed a decrease in the node degree, thereby preventing interference from other regions. Meanwhile, the left A1 showed increased node degree and enhanced integration of neural coupling with a sensory region, suggesting that the left A1 acts as a hub for stimulus detection. 

Employing the temporal regularity of stimuli is another way to manipulate implicit expectations. In this case, subjects expect the next target based on the regularity of one sequential stimulus. Here too, in anticipatory attention with no awareness of temporal regularity, alpha-band cortical links were shown to be associative: during a prestimulus interval, enhanced alpha-band functional connectivity among the intraparietal sulcus, the ventral premotor cortex, and the anterior supplementary motor cortex was observed [60]. Thus, increased alpha-band coupling could be considered to reflect the preparation for further analysis of sensory information.

2. Preceding Cue

Another experimental paradigm that manipulates expectations to investigate the prestimulus prediction is setting an explicitly cued condition [60]. ElShafei, et al. [61] showed that informative cues make participants respond faster to the target and increase the accuracy of their performance. Again, alpha-band oscillations play a major role in the analysis of relevant upcoming stimuli for anticipatory attention. Talalay, et al. [60] observed that the anticipation of auditory stimuli was accompanied by enhanced functional connectivity in the alpha band between the right lateral prefrontal cortex and the A1.

As cues are often presented in the visual modality, a correlation between alpha power in the visual cortex and behavior has also been reported. When visual cues indicated that the upcoming target was to be presented in the auditory modality, alpha activity prior to stimulus presentation was shown to increase in the visual cortex and subjects could discriminate target sounds more quickly with higher alpha power in the occipital cortex [61]. This means that stronger inhibition, represented by enhanced alpha power, in brain regions which were less relevant to the task such as the visual cortex was correlated with better behavioral performance.

Among the asymmetries between the left and right hemispheres in spatial attention to auditory stimuli, the modulation of the *right* hemisphere by the preceding cue has been revealed. For example, increased frontoparietal functional connectivity during cued attention was observed mainly in the right hemisphere [60]. In addition, alpha power was modulated only in the right auditory cortex in response to visual cues, while the left auditory cortex did not show such a modulation effect. Specifically, in the pre-target period, informative cues led to a modulation in the alpha power in the right auditory cortex, showing a relative decrease in power when the next target was anticipated in the contralateral left ear and a relative enhancement when the next target was indicated in the ipsilateral right ear [61,63]. 

#### 3.2.2. During Exposure to Stimuli

1. Pure Tone Sequences

N1-P2

Unimodal auditory click stimuli (e.g., [64]) and pure tones (e.g., [65]) can induce ERP components in the auditory cortices. Among them, the early-stage electrical organizing correlates reflecting manipulation by auditory stimulation are found in the N1 component. N1 is one of the most basic perception-related ERP and is the component of investigation in many ERP experiments, including the event-detection process of auditory processing [66,67]. There is a positive correlation between the amplitude of N1 and the spontaneous functional connectivity between bilateral Heschl’s gyruses obtained by blood oxygenation level-dependent (BOLD) stimulation [65].

Two of the selected studies have shown that the auditory N1 component or N1-P2 complex are attenuated when they are predictable, via temporal probabilistic cuing [68] or rhythmic cueing [69]. Notably, one study observed the reversing of N1 attenuation by directing attention to large perturbations. Another remarkable phenomenon is motor-induced suppression of N1. Two studies observed that self-induced and self-generated sounds induced attenuation of cortical N1 amplitude [70,71]. Generative network models of those inhibitory processes reveal internal predictive inputs from higher-order cortical areas. The suppressed N1-P2 amplitudes were driven via motor commands sent into the supplementary motor area (SMA) that is responsible for the movement planning system, from where predictive signals that convey motor commands were passed to the auditory cortex [70]. In sum, temporal predictions for specific stimuli and self-generation of sounds reduce the N1 amplitudes. In contrast, an orienting of attention to the expected stimuli (i.e., a focusing of neural resources) works toward increasing the auditory N1 amplitudes.

MMN

Mismatch negativity (MMN), which is observed 100–200 ms after the occurrence of deviations from regularity, is considered a sensitive neurophysiological metric of prediction error when external sensory inputs are matched against the formed internal statistical model [72,73,74,75]. It was recently shown that the amplitude of MMN responses fluctuates not only based on the local regularity learned through the sequential experience of the sound pattern but also on the longer timescale regularity of the length of the sequence blocks [77].

A few studies focused on the contribution of rhythmic activity toward the detection of matching and mismatching auditory events. The networks underlying prediction error responses seem to employ low-frequency neural oscillations. While Nicol, et al. [76] observed localized gamma-band connectivity changes in frontal-temporal regions during the MMN period, other studies assumed theta rhythm to be a characteristic of MMN production [73]. Recasens, et al. [78] even found the involvement of cortical–subcortical networks during mismatch sequences by showing enhanced theta and alpha coupling among the auditory cortex, hippocampus and prefrontal cortex. 

Previous studies clarified the hierarchical framework between brain regions underlying MMN generation by investigating effective connectivity through dynamic causal modeling (DCM) [74,77,79]. DCM is a universal approach to modeling underlying neuronal mechanisms, which can reveal complex relationships between the estimated activity of multiple brain regions contributing to auditory ERPs [72,77]. The work by Phillips, et al. [74] showed that bilateral inferior frontal gyruses (IFG; the prefrontal cortex) are subject to predictive signals as the underlying driving input for MMN generation. In sum, MMN reflects prediction error in auditory processing, after deviations from regularity. Behind the MMN production, the involvement of underlying oscillations was identified, and DCM models contributed to visualizing the hierarchical generative network of MMN.

P300 (P3a-P3b)

Previous findings suggest that an evoked P300 inhibits the process of a subsequent stimulus, shown by the behaviorally prolonged reaction time, decreased sensitivity and physiologically decreased amplitude corresponding to the next stimulus [144,145]. One selected recent study by Houshmand Chatroudi, et al. [81] revealed the compensatory mechanism of the subsequent inhibitory effect. They showed that the suppression of the subsequent visual P300 by the preceding auditory P300 entails a further reduction in alpha power in the visual cortex, thereby activating the visual areas to determine whether the subsequent visual stimuli are cognitively important. By employing a modified three-stimulus oddball paradigm including a second infrequent stimulus, two peaks of P300 have been reported: P3a and P3b [146,147]. P3a is the earlier and more anterior component that is primarily associated with stimulus novelty and thus is highly sensitive to unpredictable distractors that cause an involuntary reorienting of attention [148,149]. P3a is elicited by non-target stimuli and is not necessarily related to the generation of responses. In contrast, P3b is the traditional P300 peak that responds to infrequent target stimuli and is observed later in more posterior regions [150]. A recent work by Blundon and Ward [82] suggested that the ventral network and the dorsal network are the sources of P3a and P3b, respectively.

The correlation between increased task difficulty and the decreased P300 amplitude and anterior–posterior interregional phase gamma-band synchrony (GBS) has previously been identified [151]. Choi, et al. [83] discussed that GBS during the P300 epoch for target processing was stronger in the dorsal attention network, which reflects top-down processing. In contrast, the GBS for non-target processing was stronger in the ventral network, which reflects bottom-up processing. Blundon and Ward [82] also showed that these two networks were coordinated by the left middle frontal gyrus (MFG). Specifically, in tasks that require focused attention, the dorsal network sends top-down signals via the MFG to inhibit the ventral network, making it respond only to task-relevant stimuli. When an unpredictable salient input is applied, the ventral network sends bottom-up information to the dorsal network via the MFG to mediate attention to that input. Thus, the interplay between the bottom-up information and top-down modulation via attention is observed behind the P300 processing.

ASSR and binaural beat

The cortical responses that we have summarized so far such as N1, P2, MMN and P300 are elicited by auditory stimuli with short duration and long ISIs. These cortical responses are excellent for analyzing the time course immediately after stimulation. Meanwhile, one method for assessing brain responses during continuously presented sound stimuli is auditory steady-state response (ASSR), a cortical response to periodic clicks or modulated acoustic stimuli [84]. Gamma-band synchronization entrained to external 40 Hz sounds extends from the auditory cortex to the entire cerebral cortex, which has been described as reflecting the fundamental property of information integration [84,152,153]. In line with this, Schuler, et al. [85] showed that in humans, 40 Hz ASSR correlates positively with MRI-estimated cortical thickness.

One way to observe such phase synchronization by stimulating the brain with specific frequencies is binaural auditory beat stimulation. Binaural auditory inputs delivered independently into each ear with small frequency mismatch are perceived in the brain as an illusional beat, called binaural beat (BB) [154,155]. The frequency of the BB is equal to the difference between the two presented tones. The BB is of great interest because it is compatible with existing neuromeric measurement systems (EEG/MEG) and can generate modulations of internal oscillations via existing auditory pathways. 

The selected studies have observed that the frequency bands of perceived BBs do not always coincide with coherence in the sensory cortex [86,87,88]. For example, one study observed predominantly enhanced alpha-phase synchronization after listening to BBs in the delta and alpha bands [89], while another study showed that BBs in the gamma band did not increase the gamma-band power in the sensory cortex [90]. Interestingly, there is a classical consensus that the BB can affect cognition and psychophysiological states. The frequency bands that affect performance seem to vary depending on the task. For example, performance on memory tasks was enhanced by BBs in the beta band, while poorer performance was elicited by BBs in the theta band [91]. In sum, ASSR reflects oscillational entrainment to continuously presented sound stimuli, and BBsare also suitable for observing phase synchronization to auditory stimuli. However, BBs reflect binaural integration rather than entrainment.

2. Modification of Temporal Structure

Temporal associations

Both preceding cues and ISIs provide temporal associations which convey information on predictive temporal relationships between successive stimuli. Temporal expectancies are investigated by fluctuating the interval between the preceding stimulus and the target stimulus [93]. 

The temporal interval distance to the preceding stimulus is positively correlated with evoked amplitude and negatively correlated with latency [66,95,96]. In line with this, Pereira, et al. [94] showed that the amplitudes of the N1 and P2 components increased with longer ISIs, both in the repeated single tone at fixed intervals and in the oddball task with different target tones at regular intervals. The fact that the ISI effect did not differ between the two tasks suggests that this effect may be due to a common mechanism for processing repetitive stimuli in a predictable environment, such as a refractory period, instead of habituation.

Whether the regularity of ISIs has any effect on later ERP components has been investigated. Schwartze, et al. [92] revealed that pre-attentive components such as P3a and MMN were not affected by regularity manipulation, within which deviants were embedded in isochronous temporal structures or irregular contexts. Ungan, et al. [97] considered this result to be somewhat enigmatic because there were other potential factors that could mask the advantage of temporal regularity. They pointed out as a confounding factor that MMN responses become weaker when deviants occur earlier than expected in irregular contexts compared to regular contexts. By setting deviations on two axes, namely timing and pitch, they circumvented this confound. Interestingly, pitch deviances were recorded at similar MMN amplitudes both in well-timed and early timing conditions, suggesting that regular timing does not benefit the pre-attentive mechanism of auditory change detection. In contrast, P3a was significantly larger for pitch deviations with shorter ISIs. Ungan, et al. [97] argued that unlike MMNs reflecting a pre-attentive mechanism of deviance detection, this P3a result suggests a stronger involuntary attentional switch to deviance that occurred earlier than expected. The observed inconsistencies regarding P3a remain controversial.

P3b reflects top-down attention during the auditory attention task of target detection. Schwartze, et al. [92] revealed that the P3b component was larger when the target deviants were embedded in isochronous temporal structures than in irregular contexts. Beta oscillations seem to be at work in endogenous communication in target detection [62,156]. For example, Mamashli, et al. [98] employed a cued auditory attention task in which participants detected target sounds in one ear and ignored occasional novel sounds in the opposite ear. They reported stronger beta-band functional connectivity in response to the target stimuli than to the novel stimuli across the regions of interest. In sum, the modulation of the ERP components is observed in response to the manipulations of ISIs, which affects the predictive process. Later components, such as P3 and P3breflect attentional modulation of the bottom-up responses.

Rhythmic contexts and hazard rates

Rhythmic stimulation improves auditory discrimination performance, increases neural phase locking at the stimulus onset and improves EEG/MEG-based decoding compared to randomly jittered stimuli [99]. Studies have revealed that when temporal expectations are induced by rhythm, multiple mechanisms may be at work, including modulation of neuronal firing rates and the intensity and timing of oscillatory activity. Neural entrainment to rhythmically regular inputs is not only driven by the physical prominence of acoustic stimuli, but also by an endogenous generation of beats [54,55] and sensorimotor synchronization [100]. Additionally, one study has shown that brain regions responsible for the motor system are consistently involved in beat recognition even when people do not move their bodies to auditory stimuli [101,154,157]. Jantzen, et al. [100] showed that theta coupling between the pre-supplementary motor area (SMA) and the anterior cingulate cortex (ACC) increases in response to a large positive increase in tap-tone asynchrony. Following this increase in top-down control, beta-band oscillatory activity in the primary motor cortex was shown to be enhanced, resulting in the inhibition of motor cortex. 

One idea that allows for rhythmic facilitation is bottom-up entrainment to auditory rhythm. This theory is supported by the observed different electrophysiological characteristics between the two oscillations, such as the observation that a clear neural response was elicited at the first harmonic of the beat only for the on-the-beat condition, not for the off-the-beat condition [102]. In line with this, Stupacher, et al. [55] observed that the amplitude of the N1 event-related response becomes more negative with longer silent breaks after a stimulus halt. 

Another informative temporal structure is hazard rates. Hazard rates are calculated by combining the distribution of onset times with the probability of tone onset increasing over time [45]. When participants wait for the next event to occur, the probability of the stimuli occurring at the next moment increases over time. At the same time, there is a temporal distribution of the moment at which the events occur. Thus, the estimation of the tone occurring at a given moment is a combination of the distribution of onset times and the probability of tone onset increasing over time [32,45]. With this background, the work of Todorovic and Auksztulewicz [103] modeled the passage of time and the distribution of deviant stimuli separately. They confirmed the connectivity of feedback inhibition and feedforward activation between cortical areas in the hierarchical model. In contrast, for the higher levels of cortical hierarchy, the effect of the passage of time on descending connections was asymmetric, with stronger inhibition in the left hemisphere and weaker inhibition in the right hemisphere. This reversal of connectivity can find theoretical support from the seesaw-like relationship between attention and prediction represented by the early negatives mentioned above. Thus, even in the absence of temporal cues, rhythms and hazard rates can be the dynamic clues to predict the likelihood of upcoming events. Here again, the oscillatory entrainment to rhythmic stimuli and the prediction as to the timing of the upcoming stimuli are subject to top-down modulation.

Other topics

Currently, reports in the literature on the effects of other auditory factors, such as tone variation, volume and spectral variance, on neural activities are quite limited. Indeed, only four selected studies, by Kim, et al. [104] (in this section below), Wu, et al. [125], Rogenmoser, et al. [127] (the Auditory experience in conjunction with emotional responses section in the Results 3.2.2.) and Gupta, et al. [141] (the Results 3.2.3) used real music as sound stimuli. For example, Kim, et al. [104] employed real music of Mozart’s original melody and its variation and showed differences in fronto-temporal connectivity patterns when participants listened to the modified version. However, there remains the possibility that factors other than the melody could influence the differences in connectivity patterns. 

For tone frequencies, studies on frequency-specific adaptation have had an upsurge in research interest. N1 attenuation after repetitive stimulation is a well-known phenomenon in EEG research that usually refers to repetition suppression and adaptation [66]. Through adaptation, neural circuits represent statistical properties of the entire acoustic feature dimensions, such as spectral variance. For instance, Herrmann, et al. [67] demonstrated that the N1 amplitude is larger for tones that are farther from the center of the frequency spectrum. Alongside, or instead of, repetition suppression, repetition enhancement is known to occur later than repetition suppression and is therefore functionally dissociable from repetition suppression [72]. Frequency also modulates the evoked potentials when a sound begins to move after the initial adaptation phase of being stationary. Sarrou, et al. [105] investigated whether auditory motion responses are frequency specific and revealed that moving sounds with higher-frequency bands elicited higher amplitudes of the early part of the motion-onset cortical responses than those with lower-frequency bands. 

In relation to sound volume, there is emerging evidence on the modulation of neural activities. Neural coding for sound intensity is diverse, with more neuronal firings at noise above a threshold of 60 dB [107,158]. With increasing sound intensity, the latency of auditory brainstem responses was shortened and their amplitude enhanced [96]. In addition, cortical excitation patterns expanded with increasing sound intensity (for Nd and P300: [106]; for N1-P2: [107]). The early gamma-band responses also increased with increasing sound pressure, accompanied by an increase in intertrial phase-locking, which was more prominent in the active vs. passive condition [107]. The authors ascribed this enhancement in intertrial synchrony to the fact that earlier latency of the first firing can be recorded in response to high-intensity stimuli, and this conclusion is under the assumption that sound intensity acts as a bottom-up factor (as opposed to attention as a top-down factor). Thus, research on the effect of minor auditory factors like tone variation, volume, and spectral variance on neural entrainment shows the modulation of earlier ERP components and neural synchrony depending on the context.

3. Lower- and Higher-Order Functions in Representation of Auditory Objects

Top-down modulation of bottom-up auditory processing

Recent studies on short-term neuroplasticity in auditory-object representation have focused on bottom-up and/or top-down processes. For bottom-up auditory process, Luo, et al. [108] showed that when learned noise formed into meaningful auditory objects in brain networks, neuronal phase patterns in low-frequency (3–8 Hz) auditory cortical responses gradually emerged. This finding is compatible with a population-level temporal encoding mechanism based on a phase-mediated organization pattern in time. Another study focusing on bottom-up processes pursued figure–ground segregation [109]. Teki, et al. [109] showed that figure–ground segregation of sound occurs in brain regions other than the normal auditory system: that is, the planum temporal and intraparietal sulcus. Moreover, there is a line of studies attempting to distinguish the two dimensions of *segregation* and *exploration.* Farkas, et al. [110] show that *exploration* and *segregation* are linked to different functional brain networks, with the theta frequency band related to *segregation* and its constituents while the alpha and beta frequency bands to *exploration* and its constituents.

One of the most significant features that intertwine bottom-up responses (mentioned above) with top-down responses is attention—the process of prioritizing a particular stimulus or object in the physical world for processing while filtering out less significant ones. Ahveninen, et al. [111] propose a “tuning model” for segregation of relevant sounds from noise. According to this model, figure–ground perception is supported by tuning changes in neurons based on short-term plasticity in the auditory cortex. Importantly, transient tuning changes can be viewed as an interface between bottom-up and top-down processes underlying auditory attention. A cautionary note is that the attention during listening to musical pieces could vary according to the genre of music [112]. Moreover, an MEG study that addressed auditory awareness found that successful detection of two-tone pairs within complex auditory scenes relies on recurrent processing between auditory and high-order parietal cortices [113]. Another MEG study that aimed to detect the source of the auditory-evoked gamma-band response (aeGBR), which has been shown to be modulated by attention, suggested that the dorsal ACC is implicated in the attentional auditory processing [114]. Subsequently, a pioneering work by Leicht, et al. [115] more precisely verified the connectivity between the ACC and bilateral auditory cortex. Moreover, under active listening conditions using attention, the aeGBRs are more likely to be augmented for loud sounds [107].

The dynamics of the surrounding environment, which are routinely experienced in our daily life, could be reflected in task design. For instance, Choi, et al. [83] explored how varying-difficulty tasks induced changes in gamma-band neural synchronizations and showed that these changes varied considerably between target and non-target processing, in a way that matches the spatial characteristics of top-down and bottom-up attentional networks. This finding implies that, even in a single experiment, attentional modulation could vary significantly at each time point, in line with temporal shifts in task content. Furthermore, Gong, et al. [116] showed that the brain uses both asynchronous and synchronous patterns of information transmission when required to achieve rapid performance in the same single task, revealing how the brain utilizes different information processing patterns.

The latest studies have examined the relationship between audition and another prominent components of higher-order cognition, such as WM [159]. Beauchene, et al. [86] revealed that WM task performance was associated with higher power and enhanced connectivity of cortical networks in the theta band by manipulating neural synchrony via the BB. Wolff, et al. [117] examined whether auditory, as well as visual WM, depends on content-specific connectivity changes that include sensory areas. They showed that sensory processing areas were essential for the maintenance of information in WM. This result highlights the involvement of bottom-up stimulus representation in the maintenance of WM. As Wolff, et al. [117] focused on EEG electrode space analysis, the underlying source of potentially silent WM representation was somewhat elusive. Mamashli, et al. [118] further confirmed that auditory WM content is fundamentally represented in the phase-synchronization patterns of the sensory cortex using machine learning analyses of MEG recordings. In sum, lower-order auditory processing is supported by higher-order mechanisms which underlie general cognitive functions such as attention and WM. 

The aforementioned studies provide compelling evidence that EEG and MEG are useful measures to elucidate the interplay between bottom-up and top-down auditory processing. The province of EEG/MEG is still expanding; for instance, Coffey, et al. [119] presented the first measurements of the frequency-following response (FFR) using MEG and showed that the MEG equivalence of EEG–FFR can be observed. Although MEG may help reveal the interactions between higher and lower nuclei in the hierarchical auditory system [53], it is still challenging to identify the specific neural generator of FFRs. This difficulty is typical of techniques that record far-field neural responses as the sum of many single oscillatory assemblies but is also attributable to our weak understanding of the theoretical mechanism behind the bidirectional interactions between bottom-up and top-down processes. Garrido, et al. [120] addressed this problem, which will be discussed in Section 4.5.

- Auditory experience in conjunction with emotional responses

Attempts to interrogate the neural correlates of emotional processing have flourished, but investigations into the nature of music-evoked emotion itself have only just begun. One EEG study examined the neural correlates of emotional responses to music when using a larger and more varied stimulus set [122]. They provided a plausible image of sparse long-range neural connections involving several cortices and both hemispheres, which were modulated by music-provoked valence and tension. Another EEG study proposed that bilateral average activity in the beta and gamma band contributes to the best discrimination between liking and disliking judgments, that is, emotional arousal phenomena [123].

There is also a study that examined the involvement of the extended mirror neuron system [124]. They found that the mirror neuron system was highly activated during emotional vs. non-emotional perception of human action. Investigation of the neural mechanisms behind the emotional processing of music has potential clinical benefits for individuals with psychological disorders. According to Sarasso, et al. [132], the investigation of the relationship between aesthetic appreciation and knowledge achievement could be beneficial in the field of education and rehabilitation of attentional and memorization deficits. The tight connection between aesthetic appreciation and EEG indices of perceptual learning has already been empirically demonstrated by Sarasso, et al. [133].

Studying musical emotion ultimately requires considering a single piece of music as a Gestalt whole. Music is an orderly sequence of musical aspects with different complicacies. Thus, Wu, et al. [125] used the music heard in our everyday lives as auditory stimuli to reconstruct daily surroundings in the laboratory. Their findings imply that music perception requires more information processing, as well as cognitive effort. Importantly, they chose Chinese Guqin music as acoustic stimuli because Guqin music, representative of Eastern music, could provide new knowledge for current music studies, which mainly use Western music [126]. Rogenmoser, et al. [127] also used whole music excerpts with sufficient length as experimental stimuli to provide an ecologically valid prerequisite for natural music listening. The authors analyzed music-evoked emotions in terms of two affective dimensions, valence and arousal, and found that arousal appeared to be mediated by the right posterior portion of the brain, as indicated by alpha power suppression, whereas valence appeared to be mediated by the left frontal lobe, as indicated by theta power. Moreover, Tervaniemi, et al. [128] are remarkable in that they studied listeners’ brain functions in a concert-like environment rather than in a laboratory setting. While they found that theta power was enhanced by both familiar and unfamiliar improvised music, Ara and Marco-Pallares [129] revealed that neural oscillatory activities during listening to familiar vs. unfamiliar music may depend on different brain regions subserving theta connectivity patterns. The right fronto-temporal theta synchronization, which Ara and Marco-Pallares [130] had already observed as the basis of music-evoked pleasantness, increased in conjunction with reports of pleasantness, even when participants listened to unfamiliar music.

Few studies have examined the contingency reversal of classical conditioning in humans in terms of the neural network. However, such studies could lead to a better understanding of cognitive and emotional processing in the brain. Kluge, et al. [121] not only corroborated previous studies on the plasticity of human auditory responses with classical conditioning but also showed that different hierarchical levels of cortical auditory responses depend on different constraints in the flexibility of contingency reversal. Their findings suggested that cognitive and emotional influences on auditory processing are distinct. Then, it may be challenging to investigate how a certain sensation is affected by both cognition and emotion in EEG studies. Pain is one of those sensations, and it has at least been shown that pain can be controlled by brain-wave music, generated from EEG signals according to the power law of both EEG and music. For example, Huang, et al. [131] showed that orthodontic pain can be alleviated by brain-wave music, which is thought to modulate functional connectivity among different brain regions.

- Auditory plasticity relative to language processing

The neural substrate for processing musical tonal expectancy violations has been shown to overlap or mirror that of syntax processing in language. James, et al. [134] reported that the centro-posterior negativity (CPN) peaked around 400 ms after stimulus onset when primary-school children heard harmonic transgressions. This intriguing observation corroborated that CPNs, which are associated with language processing, occurred in an intra-musical context. They also suggested that, at least for infants, syntactic and semantic processing may coincide in intra-musical contexts. In fact, prelinguistic acoustic mapping of children can be influenced by attention.

Benasich, et al. [135] used non-linguistic acoustic cues that had relevance for subsequent linguistic mapping to investigate the effects of active auditory experiences compared with passive ones on infants’ neural processes. They demonstrated that active experiences increase attention and perceptual vigilance to acoustic stimuli. Therefore, top-down modulation of bottom-up auditory processing may impact the ability of the brain to decode incoming speech.

#### 3.2.3. Pre- and Post-Stimulus Period

It is now increasingly established that a relatively short period of training of subjects with no previous musical experience improved discrimination of auditory stimuli that involved neurophysiological plastic changes. The first study in the literature on plasticity research with auditory stimuli included the learning of a notched sound spectrum [28] and pitch [160,161,162]. Schulte, et al. [163] reported that after a few days of intensive training, subjects were able to discriminate newly learned pitch melodies, and this change was accompanied by a distinct increase in transient gamma-band responses and higher synchronization of cortical networks in the gamma band. Carcagno and Plack [136] have also shown that improved behavioral performance in pitch-discrimination tasks was reflected in enhanced responses.

Other studies in the literature have reported neuroplastic changes using periodic sound stimulation. Yamsa-Ard and Wongsawat [137] observed modulation of EEG power and coherence by 5 Hz BBs (theta-band enhancement). Kompus and Westerhausen [142] and Lei, et al. [143] showed that the ERP component increased after periodic acoustic stimuli, which reflects the acquisition of long-term potentiation effects. Real musical pieces, not just pitch discrimination, were presented to participants in Gupta, et al. [141]. They showed a significant reduction in functional connectivity indices before and after exposure, suggesting enhanced neuronal efficiency in the cortex.

We have already seen that orienting attention can retune neurons to segregate relevant sounds ([111]; see the Top-down modulation of bottom-up auditory processing in the Results 3.2.2.). As for animals, those trained with specific auditory stimuli exhibit an enhanced definition of tonotopic map boundaries in the A1 [160]. This finding leads us to infer that for individuals who experience attention-driven auditory plasticity, the synaptic activity is strengthened, which, in turn, increases tonotopic organization. In a similar vein, Musacchia, et al. [164] suggested that interactive auditory experiences are associated with changes in acoustic cortical mapping during the period when infants construct cortical maps for language. In this study, infants only had to learn the go/no-go procedure, but Manuel, et al. [138] focused on practicing inhibition tasks (i.e., stop-signal tasks) during auditory discrimination. They showed that improvement in task performance was associated with plastic modification in high-order fronto-basal executive networks, which regulate inhibitory control. Furthermore, Benasich, et al. [135] noted that the development of auditory mapping is more prominent with active acoustic experiences rather than passive ones during infancy. This entailed a more mature topography in the infants with interactive sessions, which supports the hypothesis that attention, even at this early age, may confer a substantial advantage. Whether such auditory perceptual enhancement generalizes beyond sensory modalities remains a topic for continued research. Lau, et al. [139] illustrate how certain aspects of neuroplasticity can develop rapidly and generalize across tasks but not across modalities. In contrast, their behavioral results provide evidence for cross-modal transfer of learning.

Another important area of research is learning-induced semantic processing in auditory discrimination. One such study is that of De Meo, et al. [140], who investigated how cortical representations of birdsongs are modulated by brief training to recognize individual species. The authors propose that the expertise in semantic discrimination of birdsong shares the same neural mechanisms with discrimination between human and animal vocalizations. In sum, as to the neuroplastic changes before and after the short-term auditory interventions, the top-down modulation on auditory processing has been investigated. Such top-down effects, including attentional modulation and semantic cognition, have been shown to be essential for neural processing at the auditory cortical level.

## 4. Discussion

The converging evidence from EEG/MEG studies with longitudinal experimental approaches using auditory stimuli argues in favor of short-term neural plasticity involved in auditory processing. We propose that the neuroplasticity associated with sound stimuli ranges from sensory processing in the auditory cortex to higher-order cognitive functions, such as attention and working memory. 

### 4.1. Inhibitory Role of Prestimulus Alpha

There is growing evidence that oscillatory activity prior to an event has a significant impact on subsequent event processing [35,43]. The selected studies show the possibility that the suppressed alpha oscillations in the prestimulus period may favor the perception (see the Prestimulus Alpha Power and Behavior section in the Results 3.2.1.). Investigations via EEG/MEG studies have led to the hypothesis of the functionally inhibitory role of alpha, concentrating the attentional resources in the task-relevant cortical regions before the target stimuli [44]. This thesis is supported by alpha-power correlation with the behavioral performance of detecting near-threshold perception and target discrimination, indexed via accuracy and reaction time [165,166]. Specifically, the accumulating evidence, including selected articles, favors the idea that a decrease in prestimulus alpha power correlates with the facilitatory processing of the following stimulus (for reviews: [167]).

Recent studies denied the prevailing theory that the increased task accuracy associated with the decrease in alpha activity is due to enhanced perceptual sensitivity, in agreement with some studies [59,168]. Instead, it has been demonstrated that these findings could result from a shift in criterion. Specifically, lowered alpha power increases baseline neural excitability and amplifies the response to both signal and noise, which results in participants predicting a liberal detection criterion with no effect on sensitivity (for review: [169]). 

Findings from auditory studies about the relationship between alpha power and perception seem inconsistent [56,170]. Some propose that neuronal sensory responses have a quadratic relationship (i.e., inverted U-shape) with neural sensitivity signals, such as prestimulus alpha power [44,171]. In those studies, which observed linear relationships, the scope of the prestimulus alpha power may have been too small to fully reveal the quadratic relationship.

### 4.2. Dilemma about Alpha Lateralization

The temporally informative structures of ISIs and temporal cues lead the participants’ expectancy and encourage them to be prepared for the upcoming stimuli (see the Interstimulus Interval and Preceding Cue in the Results 3.2.1.). In both types, alpha lateralization in the prestimulus period was observed. In the implicit expectation condition, where the length of foreperiods cannot be obviously predicted during a trial, subjects exploit the information inherent in the sound such as overall foreperiod distributions. Temporal expectancy is measured as the level of preparedness at a given point in time, so that the prediction accuracy of the next stimulus is decreased for a variable ISI [172]. The selected studies speak in favor of the inhibitory role of alpha, and its lateralization shows the aggregation of the attentional resources. In an explicitly cued condition, a specific network pattern induced by the preceding cue is reinforced by the onset of the second stimulus, and network states learned through such reinforcement would carry embedded temporal predictions [45]. 

Auditory research has focused largely on the location of the sound in space, that is, spatial attention [48,173]. Electrophysiological research of spatial attention has shown a characteristic finding of the hemispheric lateralization of alpha oscillations [62,166,174,175]. Alpha power in the parietal and sensory areas is known to increase in the hemisphere ipsilateral to the focus of attention and rather decrease in the contralateral hemisphere [165,176]. This lateralization may be boosted by introducing distractor stimuli on the unattended side [46,47,177]. ElShafei, et al. [61] strengthened the work by Weisz and Obleser [167] about a modulatory alpha enhancement in the right ear by showing that cue validity facilitated this downregulation of the right auditory cortex.

An important point recently made by Schneider, et al. [178] is the need to carefully distinguish the possibility that the mechanisms of the alpha lateralization involve either target enhancement, where the target is enhanced relative to the distractor, or distractor suppression, where the distractor is suppressed more than the target, or both functions in parallel. Thus, future studies should reconsider experimental designs either to isolate the neural source of both alpha responses, as in Wostmann, et al. [59], or to differentiate between the two by setting a neutral control condition [178].

### 4.3. Modulation of N1 by Prediction and Attention

The attenuation of the auditory N1 and N1-P2 complex in a temporally predictable context is shown in many studies, in addition to these selected studies [68,69,179,180] (the N1-P2 section in the Results 3.2.2.). A decrease in N1 amplitude is also observed with periodic oddball paradigm enforcement, of which the phenomenon is called “repetition suppression” [181]. These attenuation effects may be explained in the scheme of predictive coding (PC), which connotes that our brain minimizes prediction errors by optimizing the prediction of external sensory inputs through its internal statistical model [182]. The theory suggests that cortical responses to sensory stimuli are largely driven by the mismatch between predicted and perceived stimuli [183]. Neural processing for readily predicted stimuli is attenuated because the smaller deviations between the sensory input and the prediction results in smaller prediction errors and, hence, reduced stimulus-induced ERPs [184,185].

**Figure 4:**
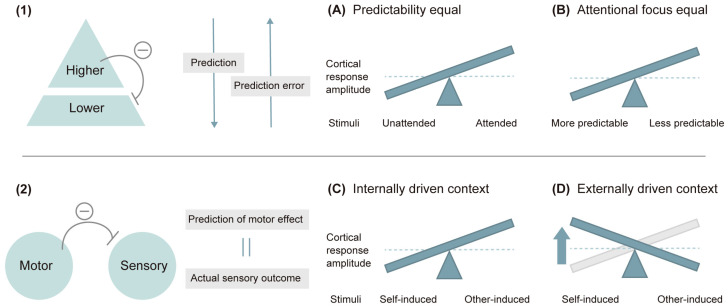
Modulatory effect of attention and prediction on cortical response suppression and enhancement. Temporal predictions of specific stimuli suppress the early negativities. In contrast, an orienting of attention to the expected stimuli works toward increasing the auditory N1 amplitudes. Note that the seesaws represent the relative relationship between the two conditions. The upper side represents higher amplitudes compared to the other side. Predictive coding theory assumes that the mismatch between sensory input and predictions is reflected in the ERPs (**1**). The following figures (**A**,**B**) show predictability and attentional focus are manipulated, respectively. The internal forward model suggests that the match of actual sensory outcome with predictions results in the inhibition of the incoming sensation (**2**). The following figures (**C**,**D**) show the predictability manipulation through internal contexts or external explicit cues, respectively, for the two conditions of self-induced and other-induced stimuli.

An orienting of attention is thought to reverse this principle: the attenuation effect can be reversed by directing attention to large perturbations that increase prediction error and are no longer attributed to an internal error [69,186]. It was observed that sounds presented to the attended ear evoke higher N1 amplitudes than those presented to the opposite ear [187] (note: this effect is sometimes absent for P2: [188]). Recent studies showed that, for two equally predictable stimuli, attentional focus increased cortical responses relative to less attentively focused stimuli (Figure 4A) [184,189]. Moreover, an unpredictable onset of stimuli relatively increased the N1 response, which the authors interpreted as a result of more attentional focus being devoted (Figure 4B) [180,190]. Thus, attention and prediction have opposite effects on cortical responses to events.

Induced attenuation of cortical N1 amplitude by self-induced and self-generated sounds is accompanied by reduced subjective sensation [191,192]. In such a motor-related paradigm, the internal forward model is often used to explain N1 suppression in response to self-induced tones [181,193]. It describes that efference copy signals (i.e., prediction) of motor commands dynamically predict the sensorial consequences of motor actions and prepare the related cortical areas to perceive the predicted sensory input (note that the term “forward” here represents the usage of the current motor command to predict the next state) [70,193]. The actual sensory outcome is then compared with the predicted effect, and if the two match, they assume that brain activity directed to the incoming sensory input is inhibited [71,194].

A key factor for motor-induced sensory attenuation is known to be stimulus predictability. In fact, a larger N1 amplitude attenuation effect in self-induced stimuli occurs when the stimuli are predictable through inferable ISIs (Figure 4C) [179,190]. A more recent study concluded that the relative N1 attenuation effect for self-induced stimuli as compared to externally triggered sounds can be reversed by the predictability equalization induced by effective temporal cues (Figure 4D), which the authors argue is the ground that the sensory attenuation depends on the relative predictability of sensory signals and the shifts of attention between selfgenerated stimuli and other-generated stimuli [195]. 

The overall results show the attenuation of N1 amplitudes induced by temporal prediction and their reversing effect by attention. Whether the N1 component is enhanced, attenuated, or unaffected may be a consequence of the net effect of these two opposite effects of attention and prediction processes [181]. Specifically, for the N1 suppression by prediction to be canceled, the additional attentional processing that enhances N1 (e.g., the expected stimuli are response-relevant) must outweigh the reductions caused by stimulus predictability. Another possibility is that the synergistic effects between attention and prediction reverse the effect of N1 suppression by prediction alone [184]. These two models are referred to as the opposition model and the interaction model, respectively, and are introduced below in the Discussion 4.5.

### 4.4. The Generation of Prediction Error Responses

In the MMN section in the Results 3.2.2, the cortical response reflecting the internal prediction error, namely MMN, and its underlying network was introduced. When a person is exposed to a new event, they automatically refer to past events to determine if it is surprising. This theory has been traditionally investigated through an oddball paradigm, in which deviant sounds are incorporated into a continuous sound stimulus pattern. This helps to learn the regularities of the sequence and infer the degree of surprise over deviations from the predictions [185,196]. It has long been suggested that when predictions are violated, the reference period of learned patterns is dynamically updated by automatic sequential learning [197]. The results of Fitzgerald, et al. [77] were in support of this hypothesis.

Previous studies using DCM have revealed that a deviance detection system employs feedforward and feedback functional connections bilaterally and inter-hemispherically among three levels of a hierarchical network: the A1, superior temporal gyrus (STG; the temporal cortex) and inferior frontal gyrus (IFG; the prefrontal cortex) [197,198]. Auksztulewicz, et al. [199] extended the finding by Phillips, et al. [74] of the role of bilateral IFGs as the driving input for MMN generation by demonstrating that IFGs themselves originate descending signals regarding the estimated predictability of sensory inputs. At the same time, they showed that IFGs play a role in optimizing the ascending prediction error. 

Compared to standard stimuli, deviant sounds seem to reduce the inhibitory intrinsic connections in the A1 and STG and the inhibitory backward connections from the STG to A1 [38]. The former is interpreted as an increased excitability of neural populations in response to a deviant sound [72]. The latter might imply disinhibition and a corresponding increase in the excitability of A1 and STG, which may contribution to the MMN [38]. Interestingly, these three functionally coupled regions are structurally connected via the auditory white-matter pathway, which was revealed by Oestreich, et al. [79] using diffusion magnetic resonance imaging (dMRI) and EEG. 

### 4.5. Contradiction about Cortical Response Dynamics and Its Solution

There seems a dichotomy in the discussion of brain responses associated with prediction errorsin MMN responses [80,199]. For N1, their amplitudes have been shown to decrease for predicted deviations (see the N1-P2 section in the Results 3.2.2.) but also to increase in some contexts where the surprise becomes predictable. However, for MMNs, the cortical response has been found to fluctuate both up and down in predictable situations. There are traditional reports for reductions in negativities after a successive presentation of identical stimuli (i.e., repetition suppression) [200,201] and a significant MMN reduction under predictability conditions [80], while Quiroga-Martinez, et al. [38] revealed that deviations in a predictable context elicited stronger MMN responses than ones in an unpredictable context. Also, larger MMNs were elicited when the deviant occurred *within* a cohesive pattern that formed a strong expectation, compared to when they occurred *between* cohesive patterns [42]. The PC model has already succeeded in explaining the enhancement of sensory signals, in terms of the synergistic operation of predictability and attention or the cancellation of the N1 suppression by attention [189]. In fact, the inconsistency of the response attenuation effect and the enhancement effect through attentional modulation was also noted in the scheme of the internal forward model [195]. They argued that in a predictable context, the model in which the brain attenuates the signal of highly predictable self-produced sounds (Figure 4C) and the model in which attentional salience to self-produced sounds increases predictive precision and induces higher cortical responses (Figure 4A) are compatible at the same time. It seems plausible to assume that the internal forward model for motor-induced sensory suppression and higher responses by enhanced attentional salience is partially explainable in terms of the PC theory [181]. There are many studies in which attention and prediction have been intertwined or conflated [202], and future studies of the auditory system should manipulate attention and prediction independently.

In order to explain the interplay between the attention and prediction which PC supposes, two theoretical models have been presented: the opposition model and the interaction model [189]. The former model posits that attention and prediction have opposing effects on neural activity, such that prediction mitigates and attention boosts neural activity, while the latter model postulates that attention and prediction interact such that neural activity is maximal for attended *and* predicted events. Garrido, et al. [120] provided empirical evidence for these models, and the opposition model better explained EEG data.

**Figure 5:**
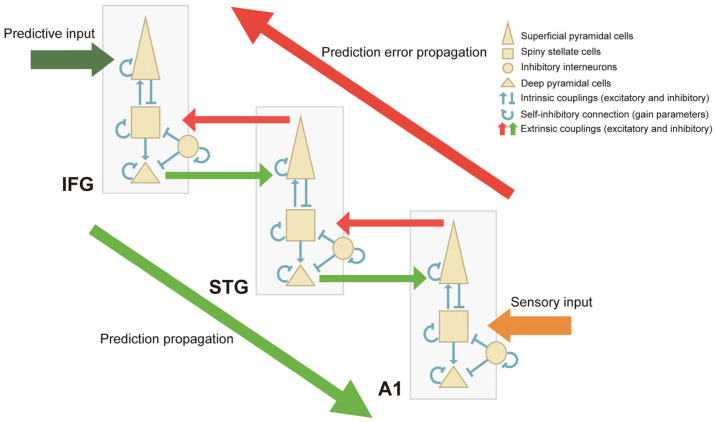
A model architecture showing the predictive coding scheme onto canonical microcircuits. This figure shows a basic model architecture of three-source DCM based on canonical microcircuits comprising four neuronal populations, as well as intrinsic connections between populations and extrinsic connections to and from different regions. The ascending extrinsic connections represent prediction errors (red arrow), whereas the descending extrinsic connections represent sensory predictions (green arrow). Each population is characterized by a gain parameter (self-inhibitory connections) encoding precision. Under DCM, the precision that should theoretically modulate the MMN amplitude is modeled by the gain level of inhibitory interneurons that synapse with the superficial pyramidal cells. The ascending prediction errors are reconciled with descending predictions from hierarchically higher areas received by the superficial pyramidal cells. In superficial pyramidal cells, prediction errors are weighted by their precision through self-inhibitory connections, which reciprocate the ensuing prediction errors. At the same time, predictions are reconciled in the deep pyramidal layers and relayed to hierarchically lower areas. References: [72,77,80,204].

Crucially, the PC theory pointed out from the beginning that prediction error may be weighted by precision [203]. Recent studies have attempted to address the contradiction by reevaluating the concept of predictive precision so that evoked responses to surprise would reflect precision-weighted prediction errors. That is, precision-weighted prediction error is the product of the multiplication of precision weight and the prediction errors [80]. In a predictable context, the prediction error is minimized, while a stimulus-driven increase in predictive precision enhances the sensitivity to upcoming sensory signals [42,204]. The apparent cortical response can therefore increase or decrease depending on the dynamics of these two forces. Thus, the need to distinguish the physiological representations between precision and prediction error arises, which was achieved by Lecaignard, et al. [80] by applying a neurocomputational dynamic modeling scheme to the auditory oddball paradigm that involved the manipulation of predictability. Their results provide further evidence for the role of gain modulation in precision weighting of prediction error. As a summary of this discussion, Figure 5 represents a model architecture of the PC scheme using a three-source DCM based on canonical microcircuits (delineated in the MMN section in the Results 3.2.2.).

### 4.6. Oscillatory Synchronization to the Presented Stimuli

In the ASSR and binaural beat section in the Results 3.2.2, the findings showed that ASSR involved the entrainment of the entire cortex. ASSR serves as an oscillator tuned to the stimulus and entrained to the phase and frequency at which the stimuli are presented [205]. Thus, the frequency of ASSR is close to the frequency of the stimulus, and maximum amplitude is observed when the stimulus is presented at a gamma-band frequency of 40 Hz [206]. It has become increasingly clear that the levels of gamma-band ASSR correlate with cognitive flexibility and attentional control measured by complex tasks and behavioral indicators of processing speed [207]. 

In contrast, there is still no consensus on whether the underlying mechanism of the BB is mediated by neural entrainment or interhemispheric coherence. Only a limited number of studies have claimed that the BB can be entrained to stimuli presented in the form of ASSR and can exhibit coherence in a frequency range close to the perceived BB frequency [208,209], and the selected studies show the discrepancy between the coherence in the sensory cortex and the frequency band of BBs. In addition, the increase in interhemispheric coherence between auditory cortices could be seen as a form of the auditory system resolving difficult binaural perceptions by increasing communication between the two cortical areas [88,210]. Taken together, these results seem to suggest that the BB phenomenon reflects binaural integration rather than entrainment. 

### 4.7. The Interplay of Bottom-Up Processing and Top-Down Modulations

Transient temporal stability emerges not only in the environment but also in the allocation of attention [211]. This notion is well represented in the theory of Dynamic Attending Theory (DAT), which connotes that predictable temporal structure guides attention and processing resources towards specific points in time [212,213]. Below, we further discuss how neural oscillations dynamically entrain to external rhythmic inputs and how attention modulates this entrainment to concurrent rhythmic stimuli, consistently with DAT [9]. Rhythmic processing is supported by the mutual relationship between top-down predictive signals and bottom-up sensory inputs [4,214]. 

**Figure 6:**
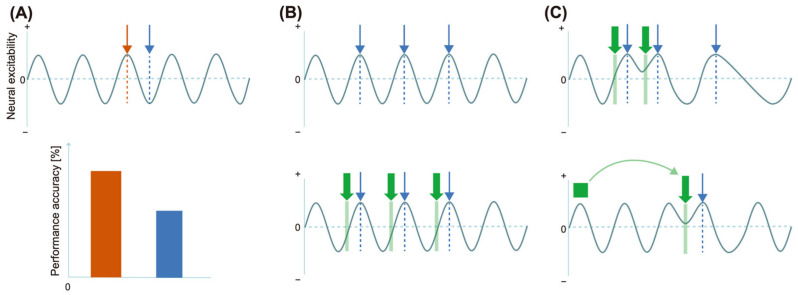
(**A**) Schematic overview of the three types of rhythmic facilitation. The blue arrows show the external stimulus inputs. Green arrows represent top-down drive from higher-order processing. A green box represents the occurrence of temporal cueing. (**A**) Spontaneous fluctuations of intrinsic oscillations: Intrinsic oscillations yield periodic alternations of low (blue) and high (red). A sensory input arriving at the high peak elicits a stronger response and leads to better performance outcomes than an input arriving at the low trough of excitability. (**B**, upper row) Neural entrainment to external rhythmically regular inputs driven by low-level stimulus features. (**B**, lower row) Top-down phase-reset modulation of neural oscillations in periodic contexts. (**C**, upper row) Neural oscillations with top-down predictive phase-reset modulations in an aperiodic context. (**C**, lower row) Neural oscillations with top-down modulations through predictive timing cueing. References: [2,9].

Selected studies have revealed the involvement of neural entrainment to rhythmic inputs and the facilitation of their processing (Figure 6B, upper row) (see the Rhythmic contexts and hazard rates section in the Results 3.2.2.). When rhythmic stimuli are presented, the brain’s responses following the temporal structure of the stimuli can also become oscillatory. This phenomenon is thought to be a neural oscillatory entrainment to the stimuli [36]. Zoefel, et al. [36] argued that these endogenous oscillatory activities were disentangled from purely sensory-driven bottom-up responses. Although Stupacher, et al. [55] failed to show conclusive evidence of prolonged oscillatory activities outlasting the stimulation (e.g., through steady-state evoked potentials), several studies did show ongoing oscillations that lasted after stimulus offset [215,216]. Collectively, the evidence suggested the endogeneity of a top-down process that enables the prediction of temporal patterns [2,4,36] (Figure 6B, lower row). 

Note that while DAT and neural entrainment theory favor periodic temporal prediction (Figure 6B, upper row) (where temporal attention is directed by the entrainment of neuronal oscillations), they cannot account for the capacity of the brain to generate temporal predictions in aperiodic stimuli. Beyond those stimulus-driven models, top-down phase-reset modulation of neural oscillations in response to anticipated events has been suggested, which is applicable in both periodic and aperiodic contexts (Figure 6B, lower row/6C, upper row) [2,9]. In short, top-down predictive drive corresponds to phase modulation of ongoing stimulus-driven neural entrainment. Another form of aperiodic temporal prediction that employs top-down-driven inputs includes temporal cueing to infer the target timing or stimulus probability of occurrence (see the Temporal associations section in the Results 3.2.2.) (Figure 6C, lower row).

According to active inference, which deviates from the free energy principle along with the PC theory, the brain minimizes prediction error by taking action so that the actual perceptual inputs can correspond to top-down predictions [183,217]. In beat perception, predictions can be updated by taking action along the beat or by establishing an internal model of the concurrent beat [218]. Behavioral evidence for prediction in beat perception can be obtained from tapping experiences along with auditory stimuli. The ability to detect and adjust the tap-tone asynchrony is measured by introducing a phase shift by advancing (i.e., negative perturbation) or delaying (i.e., positive perturbation) the stimulus interval [100]. This sensorimotor synchronization is supposedly supported by a broad network that includes the cerebellum, basal ganglia, insula and motor cortex, especially SMA and ACC [219]. A prevailing hypothesis is that different mechanisms may operate in tandem in response to positive and negative perturbations [100,220].

While subliminal (small) positive perturbations are supported by cerebellar circuits associated with accurate error correction [221,222], additional involvement of frontal motor areas has been noted for liminal (large) positive perturbations. Specifically, Jantzen, et al. [100] showed that theta coupling between pre-SMA and ACC increases in response to a large positive increase in tap-tone asynchrony. Following this increase in top-down control, beta-band oscillatory activities in the primary motor cortex were shown to be enhanced, resulting in the inhibition of the motor cortex. These two oscillatory activities may reflect the error-correction system in the increased tap-tone asynchrony that requires a subsequent tap delay or deceleration.

In contrast, there is a prevailing hypothesis that achieving sensorimotor synchronization in negative perturbation requires active anticipation of the upcoming beat [218,223]. For example, a recent study by Miyata, et al. [224] supported this perspective by showing that an individual’s predictive ability and bilateral dorsal premotor cortex activity correlate with negative tapping asynchrony. An alternative hypothesis is that the processing of synchrony errors is based on the period of the stimulus sequence or that error processing occurs within a fixed period of time following the stimulus, regardless of the interval between stimuli [220]. However, although previous studies have shown asymmetries in the perception of asynchrony and the recovery of tap synchrony after both perturbations, they have not provided insight into the broad network behind the correction mechanism for negative phase shifts [100,225].

Spontaneous fluctuations in intrinsic brain activities at a certain frequency cannot be overlooked either [9] (Figure 6A). Since the phase of ongoing fluctuations is thought to reflect the momentary excitability level, the effectivity of the stimulation process varies depending on whether the stimulus occurs in the high- or low-excitability phase [226]. Evidence in support of this theory often comes from the correlation between trial-by-trial fluctuations in behavioral performance levels and the prestimulus intrinsic phase fluctuations that depend on the predominant rhythm of the sensory system (e.g., prestimulus alpha phase in the auditory modality) [227]. Although this phenomenon does not constitute neural entrainment, it casts insight into how the neural system interacts with external rhythms.

A complementary line of research examining whether endogenous oscillations constrain the perception of stimuli concerns more complex rhythms. The process of endogenous generation of rhythmic entrainment to syncopated stimuli appears to occur at the cortical level rather than at the subcortical level, as suggested by Nozaradan, et al. [54], who observed an absence of meter-related enhancement in auditory responses at the subcortical level in complex syncopated rhythms. Another study by Stupacher, et al. [55] showed that N1 and steady-state-evoked cortical responses were similarly affected by rhythmic structure, with more complex rhythms facilitating rhythm processing in comparison with metronome drum clips. Specifically, increased rhythmic complexity was associated with greater tap-tone asynchrony and smaller N1 peak amplitudes [55,228]. This result is consistent with the N1 motor-induced suppression theory (see the N1-P2 section in the Results 3.2.2.) and the suppression of N1 with less surprise in the PC theory (see the MMN section in the Results 3.2.2.).

### 4.8. Confusion of the Terminology: Attention

A stringent question pertains to the confusion of terminology: The terms “attention” and “attentive” are loosely defined. Caution is needed in interpreting those words listed in the literature as they may have different meanings. For instance, in the Top-down modulation of bottom-up auditory processing section in the Results 3.2.2, Ahveninen, et al. [111] defined the term “attention” as the ability to select relevant information from auditory inputs in noisy environments. In contrast, Jäncke, et al. [112] asked participants to count the occurrence of specific musical aspects (pauses and changes in loudness), i.e., “attentive listening”, as they called it. An “attentionally demanding version” of auditory reaction tasks in Polomac, et al. [114] required quick and accurate responses to two out of three target tones of different pitches. Judging from these studies alone, the literature focusing on higher-order networks seems to differ in the phenomenological content of attention. It is often the case that the concept of attention is addressed in the context of higher-level neural processing but may actually vary in its mechanistic underpinnings. Important insights can be gained by studying it in terms of spontaneousness; dissociation is possible for involuntary attention and voluntary attention, that is, bottom-up attention and top-down attention [229,230]. Bottom-up attention refers to attentional guidance purely by externally driven factors in which information is selected automatically because of highly salient features of stimuli, whereas top-down attention refers to internal guidance of attention in which information is willfully picked up in the environment depending on voluntarily chosen factors [231]. Neurophysiological experiments over the past few years have shed light on the neural circuits and mechanisms of both attention systems. Additional investigation putting this dissociation into perspective is indispensable in order to fully understand the attentional influence on auditory-response variability.

### 4.9. Dissociation of Attention, Awareness and Consciousness

There is a substantial body of studies on attention which can be collectively classified as concerning auditory “figure-ground segregation” (see the Top-down modulation of bottom-up auditory processing section in the Results 3.2.2.). It is worth noting, however, that this popular research topic includes mental functions at various scales. Giani, et al. [113] described the process of detecting specific tones embedded in a multi-tone background as “auditory awareness”, which may seem to be parallel with “attention” that Ahveninen, et al. [111] mentioned. However, there is a tricky question involved in decoupling “attention” from “awareness” (or “consciousness”) conceptually. Although awareness and attention have overlapping and intertwined neural systems, accumulating evidence suggests their different functions, as well as different neural correlates. Consciousness has the function of creating a continuous and coherent picture of reality, while attention has the function of attributing relevance to the objects of thought [232]. Different degrees of awareness can be attributed to different contents of conscious experience, according to the current focus of attention, such that a more nuanced analysis of different layers of information processing will be a long-term challenge for future research on audition.

### 4.10. The Benefit of Auditory Plasticity for Language Development

As for the Auditory plasticity relative to language processing section in the Results 3.2.2, the neural substrate for processing musical tonal expectancy violations has been shown to overlap or mirror that of syntax processing in language [233]. Relatedly, James, et al. [134] corroborated that CPNs are associated not only with language processing but also with musical context, as we pointed out in the results section. The foundations of language are established in infancy: fine-grained analyses in the tens-of-milliseconds range could contribute to the decoding of the speech stream. To facilitate decoding, the developing brain constructs acoustic maps of native language sounds that enable infants to process incoming language efficiently [234]. Precisely targeted non-linguistic acoustic experiences that focus the infants’ attention on linguistically relevant environmental cues may facilitate neuroplasticity during this early developmental period [235,236,237]. Accordingly, Benasich, et al. [135] used non-linguistic acoustic cues to investigate the effects of active auditory experiences compared with passive ones on infants’ neural processes, as we pointed out in the results section. Since non-linguistic acoustic processing ability in infants robustly predicts subsequent language development [238], their reported results have significant implications not only for typical language development but also for atypical language development.

### 4.11. Confounds of Auditory Factors

To assess which specific auditory factors contribute to the changes in neural activities, we have reviewed many articles employing artificial sound sequences in which one auditory factor is designed to be clearly separated or differentiated from the others (see the Modification of Temporal Structure section in the Results 3.2.2). As artificial stimuli are of little ecological validity [39], neural processing of them does not reflect real-world settings where the brain may employ general principles that govern the processing of complex natural stimuli such as music [239]. However, the approach using natural stimuli has its disadvantages. It is difficult to set a control for auditory stimuli that resemble real music; the intercorrelations between auditory factors obscure the relative contribution of each single factor. As we noted in the Other topics section in the Results 3.2.2, four selected studies used real music as sound stimuli. Of them, the study by Kim, et al. [104] (see the Other topics section in the Results 3.2.2) employed real music of Mozart’s original melody and set its variations as control conditions. However, they lacked the rigidity of the control condition. To truly assess the effects of one musical factor, the other factors should have exactly the same pattern, designed under artificial conditions. To arbitrate the merits and demerits between artificial and real stimuli, some studies employ computer-generated auditory factors taken from real auditory environments. For example, auditory stimuli used by Cheung, et al. [240] consisted of computer-generated isochronous chord progressions which were taken from the original pop song corpus. Nonetheless, researchers should be aware that such a methodological approach ultimately cannot exclude the contribution of other musical factors to our real experiences with the music corpus, as well as the confound of the individuals’ prior musical experiences, i.e., whether they are culturally familiarized with the genre of the stimuli.

### 4.12. Sustained Post-Exposure Effects in Longitudinal Studies

Although we have shown the plastic effect induced by short-term auditory interventions in the Results 3.2.3, studies that address lasting neuroplastic changes after auditory exposure are very scarce. One such example is Lau, et al. [139], which assessed the maintenance of the EEG responses thirty days after the training. They observed that the differences among participant groups trained with three different tasks were maintained for thirty days for steady-state visually evoked potentials, but not for ASSR. How long the post-exposure effects can be observed in longitudinal studies is a matter of investigation. Measuring neural activities after exposure to auditory stimuli enables us to explore the persistence of neuroplasticity and brain network adaptability over time.

### 4.13. Dynamism of Short-Term Neural Oscillations Influenced by Various Factors

It is also important to keep in mind that attentional modulation can be exercised differently, not only from person to person but also in the same person at different times. For example, any temporal correlations between successive events, which is a hallmark of temporal-expectation studies, can greatly skew the interpretation of their findings. Thus, future avenues of research need to elucidate the details of temporal shifts of attention against a backdrop of environmental variability. As a matter of fact, attention is a continuous and sequential processing of information. The dynamism of short-term neural oscillations, which is the emphasis of this paper, is likely mediated by attentional dynamism over the course of a given auditory task. It is even possible that humans attentively perceive different sound stimuli in a common temporal framework, but how the various attentional mechanisms contribute to this overall framework remains to be tested. What complicates the issue are signals related to the sensory conditions of the body. Evidence suggests that body signals such as heart rates and respiration rates could influence intrinsic brain activities based on dynamically changing brain–body interactions [241,242]. The discussion described thus far is certainly a field ripe for investigation using EEG/MEG, which are potent tools to measure brain function with high temporal resolution. The reviewed literature has provided a detailed window into how EEG/MEG can break ground in understanding perceptual and cognitive auditory processing.

## 5. Conclusions

The converging evidence from EEG/MEG studies highlights the changes in neural oscillations associated with short-term auditory interventions. Recent advances in the growing research area of the neural basis of temporal expectation have revealed that even in the predictive period prior to a target, the prestimulus alpha oscillations dynamically fluctuate depending on the context, which influences the target processing. Many studies on temporal predictions show that various ERP components are modulated in a way that implements the PC scheme. Importantly, the reviewed literature suggests that short-term neuroplasticity is supported in part by higher-order mechanisms which underlie general cognitive functions. Bottom-up and top-down auditory processing are distinct and separate, albeit strictly intertwined, processes present in audition. Post-exposure effects of such neuroplasticity and the chronological dynamics thereof are open for future work. Research efforts also need to be invested in clarifying terminology by distinguishing between distinct neural activities that are often lumped together as reflecting “attention” but may actually vary in their mechanism. By observing neural activities in a carefully controlled manner and revealing the behavioral consequences on perception or cognition, we will likely be able to provide a more comprehensive account of brain function in our sound-filled world and, ultimately, what is driving perception and cognition.

## Figures and Tables

**Table 2:** A summary of articles included in the final analysis. The discussion in this paper is drawn from these papers that were incorporated into the review in the final analysis. Articles cited in more than one section are mentioned in this table in the section where they appear for the first time.

Reference Number	Content of the Auditory Stimuli	Tasks during the Experiment and Paradigms	Number of Participants	Stimulus More Than a Day	Recording	Major Findings
3.2.1. Prestimulus effects						
1. Prestimulus alpha power and behavior						
[56]	Sequence patterns comprising pure tones	Tone pattern indication task	17	-	EEG	Different prestimulus EEG phase between correct and incorrect trials
[57]	A short target sound within the background sound	Target sound detection task	12	-	EEG	The dependence of the chance of target detection on power and phase of theta-band oscillations before target
[58]	White noise bursts presented near hearing threshold with various inter-trial intervals	Near-threshold detection task	19	-	MEG	A decrease in alpha power in the auditory cortex prior to conscious percepts
[59]	Two identical sine tones	Pitch discrimination and confidence rating	17	-	EEG	A negative link between prestimulus alpha power and decision confidence
2. Interstimulus interval						
[60]	Presentation of two frequencies, whose temporal order was explicit through a cue or learned implicitly	Temporal order judgment task	24	-	EEG	Enhanced functional links in implicit anticipation
3. Preceding cue						
[61]	Target sounds with two different frequencies preceded by a visual cue as to the spatial location	Spatial attention task	14	-	MEG	An asymmetrical modulation of alpha power within the right AC1, depending on the cued side
[62]	A target sound and a distractor sound presented simultaneously on opposite ears, preceded by an auditory cue on either ear	Spatial attention task	11	-	MEG	Alpha lateralization in a right-lateralized network in response to cue validity and side-related acoustic stimulation
[63]	Standard tones and target tones that changed the modulation frequency, preceded by a visual cue to shift the focus of either ear	Spatial attention task	15	-	MEG	A stronger alpha power increase for the attend-right condition in the right AC1
3.2.2. During exposure to stimuli						
1. Pure tone sequences						
● N1-P2						
[64]	Auditory click stimuli	Listening	6	-	EEG	Evoked ERPs over both the auditory and visual cortex by unimodal click stimuli
[65]	Identical auditory stimuli consisting of brief pure tones	Listening	19	-	EEG and fMRI	Positive correlation with N1 magnitude of spontaneous functional connectivity between bilateral Heschl’s gyruses
[66]	Pure tones with varying frequency separation and stimulus onset asynchrony	Oddball-like paradigm	15	-	EEG	Decrease in stimulus-specific adaptation with the increase in stimulus onset asynchrony
[67]	Random tone sequences varying in spectral variance	Detecting deviants vs. ignoring stimuli	20	-	EEG	Largest frequency-specific neural responses on the N1 component
[68]	Sounds with onsets that were either predicted by a visual cue or unpredicted	Attending or unattending intervals	37	-	EEG	An N1 enhancement effect for attended sounds and an N1 suppression effect for predicted sounds
[69]	Regular and irregular rhythmic sequences of tones	Responding to deviants in the attended ear	34	-	EEG	Attenuated N1 for tones when rhythm predictability was high and was enhanced by attention to tones
[70]	A self-generated or externally generated tone	Indicating onset of the motion or tone	39	-	EEG	Suppressed N1–P2 complex when the tone was self-generated compared to externally generated
[71]	A single marimba tone	Self-generation of tones vs. listening	24	-	EEG	An attenuated N1 component for self-generated tones as compared to externally generated tones
● MMN						
[72]	Sine wave tones delivered at six possible carrier frequencies	Mismatch paradigm	20	-	MEG	Mismatch responses to frequency deviants being modulated by temporal attention strongly
[73]	Randomly ordered sequences of two tones	Oddball paradigm	13	-	EEG	Strong theta-band phase synchrony between the frontal and temporal areas during deviant processing
[74]	Standard sinusoidal tones and deviant tones that differed in duration, frequency, intensity, location or a silent gap	Multiple mismatch paradigm	11	-	MEG	Prediction error responses in bilateral AC1, STG and lateral prefrontal cortex for deviations
[75]	Melodies in either guitar or marimba timbre	Passive listening of oddball paradigm	38	A total of a few hours over a week	EEG	A larger negative response in auditory areas for tones previously experienced during exposure
[76]	Standard frequency tones interspersed randomly with deviant frequency trials	Passive listening of mismatch paradigm	16	-	MEG	Increased interlobar, long-distance synchronization during the MMN time epoch for deviants
[77]	Two different tones each becoming deviants in different blocks	Automatic sequential learning	19	-	EEG	Errors within the first block type exerting influence on the updating of longer timescale predictions
[78]	Sound sequences containing predictable repetitions and order manipulations	Orthogonal auditory one-back task	17	-	MEG	Involvement of theta-band oscillations for prediction-error generation in cortical–subcortical networks
[79]	A stream of sounds with log-frequencies and different standard deviations	Auditory frequency oddball paradigm and a simultaneous visual n-back task	89	-	EEG and MRI	The dynamics of auditory mismatch responses being interconnected by auditory white-matter pathways
[42]	Eight tones presented in two different four-tone patterns	Passive listening of statistical learning of melodic patterns	10	-	EEG	Stronger signal strength when cohesive patterns were violated
[38]	Simple melodies consisting of a repeated pitch pattern and novel melodies with less repetitive structure	Listening	40	-	MEG and MRI	Larger MMNm responses for pitch deviants in highly predictable compared to less predictable melodies
[80]	Repeating 42-tone pattern following the deterministic incrementing rule or pseudo-randomly assigned tones	Passive listening of oddball paradigm with predictability manipulation	20	-	EEG, MEG and MRI	Adaptive learning of surprise with larger integration of past information in the context of expected surprises
● P300 (P3a-P3b)						
[81]	Two sinusoidal tones assigned as target and standard stimuli	Auditory followed by visual oddball tasks	24	-	EEG	Inhibitory effect of auditory P300 influencing second target processing
[82]	Two types of runs consisted of two tones with different frequency	Target detection in an oddball paradigm	17	-	EEG	Ventral Attention Network and Dorsal Attention Network as the neural generators of P3a and P3b, respectively
[83]	Three tones with different frequencies	Target discrimination in an oddball paradigm	15	-	EEG	Difficulty-related changes in inter-regional gamma-band synchrony in target/non-target processing
● ASSR and binaural beat						
[84]	Amplitude modulated white noise on either ear	Passive listening	19	-	EEG	Successful location of subcortical and cortical sources of ASSR
[85]	Binaural exposure of 40 Hz amplitude modulated auditory tones	Auditory-driven gamma synchronization paradigm	52	-	MEG and MRI	Gamma synchrony of the entire cortical mantle driven by auditory stimulation in the gamma range
[86]	Acoustic stimulation conditions (none, pure tones, classical music, 5 Hz BBs, 10 Hz BBs and 15 Hz BBs)	Passive listening and N-back verbal working memory task	34	-	EEG	15 Hz BBs affecting cortical network properties
[87]	7 Hz and 40 Hz BBs and monaural beats	Passive listening and mood self-report	16	-	EEG	Cross-frequency activity elicited by BBs
[88]	10 Hz and 4 Hz BBs and corresponding monaural beats	Listening (expt. 1)	9 (expt. 1)	-	EEG	Enhanced alpha-band synchrony between auditory cortices during auditory stimulation by BBs
[89]	Non-binaural beats and BBs with frequency varying from 1 Hz to 48 Hz	Passive listening and rating pleasantness after exposure	32	-	EEG	Enhanced alpha-phase synchronization after listening to BBs in the delta and alpha bands
[90]	Pink noise, 40 Hz BBs and 40 Hz monaural beats	Selective attentional task	25	-	EEG	No occurrence of neural entrainment by 40 Hz BBs
[91]	White noise and 20 Hz BBs or 5 Hz BBs	Free recall task and recognition task	32	-	EEG	Improved free recall and recognition by beta-frequency BBs
2. Modification of temporal structure						
● Temporal associations						
[92]	An isochronous sequence and a random oddball sequence, varying the ISI duration	Deviant counting	24	-	EEG	Smaller P3b for deviant tones embedded in irregular temporal structure
[93]	A standard stimulus and a deviant stimulus consisting of 5 pure-tone sequences with various ISIs	Delayed matching-to-sample task	20 (Expt. 2)	-	MEG	Increased alpha power in temporal auditory regions with longer delay durations
[94]	Identical pure tones or standard and deviant pure tones	Single-tone task and an auditory oddball task	22	-	EEG	Enhanced N1 and P2 amplitudes with longer ISIs
[95]	Pure tones delivered monaurally at four different levels of stimulus onset asynchrony	Passive listening	20	-	EEG	Increased amplitude and decreased peak latency with increasing stimulus onset asynchrony
[96]	Two chirp trains applied concurrently at different repetition rates	An analog to forward-masking paradigm	11	-	EEG	Decreased amplitudes with decreasing distance to the preceding stimulus of the other stimulus train
[97]	Standard tones and deviant tones which differed in pitch and/or onset timing	Passive listening of mismatch paradigm	10	-	EEG	Larger P3a for pitch deviations with shorter ISIs
[98]	A buzzer cue, a target harmonic sound, which were sometimes replaced with task-irrelevant novel sounds	Cued auditory attention task	13	-	MEG	Stronger beta-band functional connectivity in response to the target stimuli than to the novel stimuli
● Rhythmic contexts and hazard rates						
[99]	A pure-tone acoustic stream interleaved with chords presented in a rhythmic or jittered way	Auditory discrimination task	23	-	EEG and MEG	Improved neural decoding of targets and distractors by rhythmic expectation
[54]	Rhythmically regular or syncopated sequences of a repeated non-harmonic chord with three partials	Tapping task	20	-	EEG	Increased amplitudes at meter-related frequencies compared to meter-unrelated frequencies
[55]	Drum clips with different rhythmic structures interrupted by silent breaks	Tapping task or passive listening	14	-	EEG	More negative N1 amplitude for metronome than for rhythmic sequences
[100]	Auditory metronome with delayed or advanced phase shift and with large or small perturbations	Sensorimotor synchronization task	16	-	EEG	Theta coupling between pre-SMA and ACC increases in response to a large positive tap-tone asynchrony
[101]	Multiple musical rhythmic patterns by manipulating note values in beats while keeping time signature	Reporting experienced arousal and valence	18	-	EEG	Emotional changes associated with the alpha-band connectivity alterations in the fronto-central connections
[102]	A single pop song with a super-imposed bassoon sound either lined up or shifted away from the beat	Passive listening	98	-	EEG	A clear neural response elicited at the first harmonic of the beat only for the on-the-beat condition
[103]	Two standard pure tones with various ISIs and a deviant stimulus which replaced either of a standard stimulus	Deviant detection in a two-tone paradigm with various ISIs	25	-	MEG	The asymmetric effect of the passage of time on descending connections
● Other topics						
[104]	A theme with an original melody of Mozart and its significant variations	Passive listening	25	-	MEG	Increased beta connectivity with modified melody compared to the original melody
[105]	Combinations of two sounds with a low to moderate and a high frequency range, either stationary or moving	Modality-change detection in a delayed motion-onset sound paradigm	14	-	EEG	Larger amplitudes of motion responses elicited by stimuli with high frequency range
[106]	Rhythmically regular and an irregular music presented with an intermittent and continuous type of stimulation	Target detection in an auditory monitoring task	22	-	EEG	Smaller P300 amplitude during the continuous and regular compared to the intermittent condition
[107]	Pure 1000 Hz sine tones presented at three systematically varied sound intensities	A forced-choice discrimination task or passive listening condition	22	-	EEG	Stronger GBRs and enhanced phase locking under the active condition compared with passive listening
3. Lower- and higher-order functions in representation of auditory objects						
● Top-down modulation of bottom-up auditory processing						
[108]	A noise sample generated by concatenating three identical noise segments or a running noise	Noise type detection in an unsupervised noise memory paradigm	13	-	MEG	The establishment of low-frequency oscillatory phase patterns in auditory neuronal responses during learning new acoustic representations
[109]	Signals comprised of a sequence of brief broadband chords containing random pure tone components	Performing auditory figure-ground segregation during a visual task	16	-	MEG	Neural sources underlying bottom-up-driven figure-ground segregation
[110]	Auditory streaming stimuli with cyclically repeating patterns	Reporting perception of four categories of auditory patterns	60	-	EEG	Functional brain networks underlying idiosyncratic switching patterns in multi-stable auditory perception
[111]	Two asynchronous standard-tone streams presented to different ears, in separate blocks with or without notch-filtered white-noise masking	Performing a selective attention task	10	-	MRI, fMRI, MEG and EEG	Short-term tuning changes in neurons that support segregation of relevant sounds from noise
[112]	An electronic pop song and a classical musical piece	Attentive and passive listening of musical pieces	30	-	EEG	Different neural activations depending on the direction of attention
[113]	A pair of target tones embedded within a multi-tone mask	Detecting a pair of tones embedded within a multi-tone background	21	-	MEG	Recurrent processing between auditory and higher-order parietal cortices in complex auditory scenes
[114]	Tones with timbres of three different pitches	Performing a choice reaction task	13	-	MEG	The involvement of dACC in the effortful processing of auditory stimuli
[115]	Tones of three different pitches	Performing a choice reaction task	28	-	EEG and fMRI	Top-down influence of the ACC on the AC executed by means of gamma synchronization
[116]	Four pure-tone stimuli with different pitches and intensities	Performing pitch and intensity go/no-go assignments	24	-	EEG	Cognitive plasticity during learning that involves transformation of asynchronous/synchronous processing pattern
[117]	Structured visual stimuli and pure tones	Performing a visual and auditory working memory task	47	-	EEG	The extent to which sensory processing areas are essential for the maintenance of information in working memory
[118]	Six ripple velocities separated by their just-noticeable differences	Performing a working memory task in a retro-cueing paradigm	20	-	MEG	Synchronization patterns across auditory sensory and association areas that support neuronal coding of auditory WM content
[119]	Pure tones with different frequencies	Fine pitch discrimination	20	-	MEG	The neural origins of the FFR
[53]	Two pure tones with a frequency of 89 and 333 Hz	Watching a silent movie whilst ignoring auditory stimulation	21	-	MEG and EEG	Neural generators of the frequency-following response elicited to stimuli of low and high frequencies
[120]	Independent streams of white noise concurrently in each of the two ears	Detecting brief gaps in noise streams	21	-	EEG	Opposing effects of attention and expectations within a fronto-temporal network engaged in sensory prediction errors
● Auditory experience in conjunction with emotional responses						
[121]	Random sequences of high or low tones	Listening auditory stimuli with classical conditioning and contingency reversal	19	-	MEG	Plasticity of auditory cortex responses when sounds are paired with shock in a classical contingency
[122]	Excerpts from film scores spanning a variety of styles	Reporting music-evoked emotional responses	31	-	EEG	Neural correlates of musical stimuli-induced emotion, such as pre-frontal cortex asymmetry
[123]	Musical excerpts from four common musical genres	Reporting liking of music	9	-	EEG	Larger amplitudes of motion responses elicited by stimuli with high frequency range
[124]	Sounds of a Tibetan singing bowl	Action–perception cycle of sound making	32	-	MEG	Brain processes underlying perception after learning a new association between a sound and the action for making that sound
[125]	Three pieces of Guqin music	Listening in varying auditory surroundings	16	-	EEG	Increase in functional connectivity as well as a more random network structure in the alpha2 band during music perception
[126]	Guqin music and pink noise	Listening to auditory stimuli in various conditions	20	-	EEG	Increased connectivity and topological change in functional networks with an enhancement of small-world attributes
[127]	A pool of 40 various musical excerpts	Reporting induced emotional responses	22	-	EEG	Independent component processes underlying emotions during natural music listening
[128]	Trio live performance	Rating improvisation, attractiveness and emotion in concert-like auditory surroundings	16	-	EEG	Theta activity reflecting the presence of improvisation in the performances
[129]	Experimental excerpts taken from sixty musical fragments	Reporting familiarity of music	22	-	EEG	Different theta connectivity patterns underlying pleasantness evoked by familiar and unfamiliar music
[130]	Experimental excerpts taken from sixty musical fragments	Reporting music-evoked pleasantness	25	-	EEG	Fronto-temporal theta phase synchronization underlying music-evoked pleasantness
[131]	Brainwave music	Psychotherapy in pain management	36	-	EEG	Improved functional connectivity among different brain regions and brain regularity induced by listening to brainwave music
[132]	More and less consonant chords and intervals	Memorizing chords and evaluating the beauty of the intervals	60 (Expt. 1), 22 (Expt. 2)	-	EEG	A relationship between aesthetic appreciation and implicit learning dynamics, as well as memorization
[133]	More and less consonant fifths and dissonant tritones with two different frequencies	Performing aesthetic judgment and detection tasks	26	-	EEG	A positive correlation between aesthetic appreciation and perceptual learning
● Auditory plasticity relative to language processing						
[134]	Musical pieces with a regular ending or a harmonic transgression at closure	Musical violation discrimination	16	-	EEG	A specific neural correlate of musical violation expectation in primary-school children
[135]	Modulated nonspeech stimuli	Performing a go/no-go looking task	49	-	EEG	Prelinguistic acoustic mapping affected by active auditory exposure
3.2.3. Pre- and Post-stimulus period						
[136]	Different complex tone stimuli	Pitch discrimination	27	An hour for 10 days	EEG	Subcortical plasticity induced by pitch discrimination training
[137]	Piano music mixed with a 5 Hz (theta band enhancement) BB	Listening	7	5 min a day for a week	EEG	After seven days of training, modulation of the absolute power, relative power and coherence
[138]	Band-pass noise bursts	Performing a stop-signal task	13	-	EEG	Plastic modifications within inhibitory control networks
[139]	Band-pass-filtered harmonic complexes	Discriminating auditory fundamental frequency, amplitude modulation rate or visual orientation	40	30 min a day for 6 days	EEG	Sustained cortical and subcortical measures of auditory and visual plasticity following short-term perceptual learning
[140]	Songs of different bird species	Auditory semantic categorization	19	-	EEG	The cortical representation of birdsongs modulated by brief training to recognize individual bird species
[141]	Indian classical music	Mood assessment before and after listening	20	-	EEG	On exposure to music, reduced information flow in long-distance connections
[142]	A standard sinusoidal tone alternating with two tones before/after a stimulation with a deviant tone continuously at 13 Hz	Mismatch paradigm and LTP-like stimulation	21	-	EEG	Increased amplitude of the negative-going MMN wave led by the LTP-like stimulation
[143]	Probe blocks of pure-tones, narrow-band noises and white noises or their tetanic presentation	A tetanic-stimulation paradigm	10	One day rest between conditions	EEG	Higher post-tetanus amplitude of the N1 component in the tetanus condition than the pre-tetanus state

## Appendix A

**Table A1:** PRISMA-ScR Checklist.

Item	Item	Prisma-ScR Checklist Item	Section of This Review
Title	1	Identify the report as a scoping review.	Title
Structured summary	2	Provide a structured summary that includes (as applicable): background, objectives, eligibility criteria, sources of evidence, charting methods, results and conclusions that relate to the review questions and objectives.	Abstract
Rationale	3	Describe the rationale for the review in the context of what is already known. Explain why the review questions/objectives lend themselves to a scoping review approach.	Introduction
Objectives	4	Provide an explicit statement of the questions and objectives being addressed with reference to their key elements (e.g., population or participants, concepts and context) or other relevant key elements used to conceptualize the review questions and/or objectives.	Introduction
Protocol and registration	5	Indicate whether a review protocol exists; state if and where it can be accessed (e.g., a Web address); and if available, provide registration information, including the registration number.	Search strategy
Eligibility criteria	6	Specify characteristics of the sources of evidence used as eligibility criteria (e.g., years considered, language and publication status), and provide a rationale.	Selection criteria
Information sources	7	Describe all information sources in the search (e.g., databases with dates of coverage and contact with authors to identify additional sources), as well as the date the most recent search was executed.	Search strategy
Search	8	Present the full electronic search strategy for at least 1 database, including any limits used, such that it could be repeated.	Search strategy
Selection of sources of evidence	9	State the process for selecting sources of evidence (i.e., screening and eligibility) included in the scoping review.	Selection criteria
Data charting process	10	Describe the methods of charting data from the included sources of evidence (e.g., calibrated forms or forms that have been tested by the team before their use and whether data charting was done independently or in duplicate) and any processes for obtaining and confirming data from investigators.	Classification of selected articles
Data items	11	List and define all variables for which data were sought and any assumptions and simplifications made.	Characteristics of the interventions in the selected articles
Critical appraisal of individual sources of evidence	12	If done, provide a rationale for conducting a critical appraisal of included sources of evidence; describe the methods used and how this information was used in any data synthesis (if appropriate).	Screening of articles
Synthesis of results	13	Describe the methods of handling and summarizing the data that were charted.	Classification of selected articles
Selection of sources of evidence	14	Give numbers of sources of evidence screened, assessed for eligibility and included in the review, with reasons for exclusions at each stage, ideally using a flow diagram.	Screening of articles
Characteristics of sources of evidence	15	For each source of evidence, present characteristics for which data were charted and provide the citations.	Screening of articles
Critical appraisal within sources of evidence	16	If done, present data on critical appraisal of included sources of evidence (see item 12).	Screening of articles
Results of individual sources of evidence	17	For each included source of evidence, present the relevant data that were charted that relate to the review questions and objectives.	Individual study results and synthesis
Synthesis of results	18	Summarize and/or present the charting results as they relate to the review questions and objectives.	Individual study results and synthesis
Summary of evidence	19	Summarize the main results (including an overview of concepts, themes and types of evidence available), link to the review questions and objectives and consider the relevance to key groups.	Discussion
Limitations	20	Discuss the limitations of the scoping review process.	Discussion
Conclusions	21	Provide a general interpretation of the results with respect to the review questions and objectives, as well as potential implications and/or next steps.	Conclusion
Funding	22	Describe sources of funding for the included sources of evidence, as well as sources of funding for the scoping review. Describe the role of the funders of the scoping review.	Acknowledgments

**Table A2:** RoBANS of the screened articles (the green and red color shows low and high risk of bias, respectively. The yellow color shows unclear risk of bias).

References	Selection of Participants	Confounding Variables	Measurement of Exposure	Blinding of Outcome Assessments	Incomplete Outcome Data	Selective Outcome Reporting
[56]	●	●	●	●	●	●
[57]	●	●	●	●	●	●
[58]	●	●	●	●	●	●
[59]	●	●	●	●	●	●
[60]	●	●	●	●	●	●
[61]	●	●	●	●	●	●
[62]	●	●	●	●	●	●
[63]	●	●	●	●	●	●
[64]	●	●	●	●	●	●
[65]	●	●	●	●	●	●
[66]	●	●	●	●	●	●
[67]	●	●	●	●	●	●
[68]	●	●	●	●	●	●
[69]	●	●	●	●	●	●
[70]	●	●	●	●	●	●
[71]	●	●	●	●	●	●
[72]	●	●	●	●	●	●
[73]	●	●	●	●	●	●
[74]	●	●	●	●	●	●
[75]	●	●	●	●	●	●
[76]	●	●	●	●	●	●
[77]	●	●	●	●	●	●
[78]	●	●	●	●	●	●
[79]	●	●	●	●	●	●
[42]	●	●	●	●	●	●
[38]	●	●	●	●	●	●
[80]	●	●	●	●	●	●
[81]	●	●	●	●	●	●
[82]	●	●	●	●	●	●
[83]	●	●	●	●	●	●
[84]	●	●	●	●	●	●
[85]	●	●	●	●	●	●
[86]	●	●	●	●	●	●
[87]	●	●	●	●	●	●
[88]	●	●	●	●	●	●
[89]	●	●	●	●	●	●
[90]	●	●	●	●	●	●
[91]	●	●	●	●	●	●
[92]	●	●	●	●	●	●
[93]	●	●	●	●	●	●
[94]	●	●	●	●	●	●
[95]	●	●	●	●	●	●
[96]	●	●	●	●	●	●
[97]	●	●	●	●	●	●
[98]	●	●	●	●	●	●
[103]	●	●	●	●	●	●
[99]	●	●	●	●	●	●
[54]	●	●	●	●	●	●
[55]	●	●	●	●	●	●
[100]	●	●	●	●	●	●
[101]	●	●	●	●	●	●
[102]	●	●	●	●	●	●
[104]	●	●	●	●	●	●
[105]	●	●	●	●	●	●
[106]	●	●	●	●	●	●
[107]	●	●	●	●	●	●
[108]	●	●	●	●	●	●
[109]	●	●	●	●	●	●
[110]	●	●	●	●	●	●
[111]	●	●	●	●	●	●
[112]	●	●	●	●	●	●
[113]	●	●	●	●	●	●
[114]	●	●	●	●	●	●
[115]	●	●	●	●	●	●
[116]	●	●	●	●	●	●
[117]	●	●	●	●	●	●
[118]	●	●	●	●	●	●
[119]	●	●	●	●	●	●
[53]	●	●	●	●	●	●
[120]	●	●	●	●	●	●
[121]	●	●	●	●	●	●
[122]	●	●	●	●	●	●
[123]	●	●	●	●	●	●
[124]	●	●	●	●	●	●
[125]	●	●	●	●	●	●
[126]	●	●	●	●	●	●
[127]	●	●	●	●	●	●
[128]	●	●	●	●	●	●
[129]	●	●	●	●	●	●
[130]	●	●	●	●	●	●
[131]	●	●	●	●	●	●
[132]	●	●	●	●	●	●
[133]	●	●	●	●	●	●
[134]	●	●	●	●	●	●
[135]	●	●	●	●	●	●
[136]	●	●	●	●	●	●
[137]	●	●	●	●	●	●
[138]	●	●	●	●	●	●
[139]	●	●	●	●	●	●
[140]	●	●	●	●	●	●
[141]	●	●	●	●	●	●
[142]	●	●	●	●	●	●
[143]	●	●	●	●	●	●
